# RSPO4 exerts tumor suppression through antagonizing canonical and non-canonical Wnt signaling

**DOI:** 10.7150/ijbs.124734

**Published:** 2026-01-01

**Authors:** Zhenfang Du, Lili Li, Xingsheng Shu, Chen Li, Qian Tao

**Affiliations:** 1 Department of Genetics and Developmental Biology, School of Medicine, Southeast University, Nanjing, Jiangsu 210003, China; 2 Cancer Epigenetics Laboratory, Department of Clinical Oncology, State Key Laboratory of Translational Oncology, Sir YK Pao Center for Cancer, The Chinese University of Hong Kong, Shatin, Hong Kong, China.; 3 School of Medicine, Health Science Center, Shenzhen University, Shenzhen 518060, China.

**Keywords:** *RSPO4*, DNA methylation, Wnt signaling, proliferation, metastasis, stemness

## Abstract

R-spondins are a family of four secretory proteins reported to be Wnt agonists. Among them, R-spondin 4 (RSPO4) is unique, with the lowest binding affinity towards ZNRF3/RNF43 and the lowest efficacy in regulating Wnt/β-catenin signaling. RSPO4 has been shown to play important roles in nail development, liver fibrogenesis and periodontitis, while its role in cancerous context remains largely unknown. In this study, we performed multi-omic analysis on transcriptional expression and methylation pattern of *RSPO4*. *In vitro* cell-based assays were performed to evaluate the functionality of RSPO4. Through cancer epigenomics, we identified RSPO4 as a candidate tumor suppressor with tumor-specific epigenetic inactivation. We further found that *RSPO4* is readily expressed in human normal tissues, but frequently downregulated or silenced in multiple cancer types due to its promoter CpG methylation. Functional studies showed that RSPO4 inhibited tumor cell proliferation, migration, invasion and stemness, through antagonizing canonical and non-canonical Wnt signaling. Mechanistically, RSPO4 exerted suppressive effects on Wnt signaling in an LGR4/5- and ZNRF3- dependent manner, through promoting LRP6 degradation and ZNRF3 stabilization. Our study revealed a novel role of RSPO4 as a tumor suppressor through antagonizing Wnt signaling, which provides important implications for development of diagnostic biomarkers and targeted therapy.

## Introduction

Abnormal inactivation of tumor suppressor genes (TSGs) by promoter CpG methylation is pertinent in almost every step of cancer development and metastasis 1. Silencing of TSGs by promoter CpG methylation leads to abnormal activation or potentiation of target signaling pathways, which subsequently confers tumor cell survival and proliferative advantages during cancer progression 2. This abnormality leads to more complex and integrated changes in signaling regulation 2. Thus, identification of novel aberrantly methylated genes offers important insights into the molecular mechanisms of cancer pathogenesis 1, 3.

Abnormal activation of Wnt signaling through disruption of signaling regulators through promoter CpG methylation frequently contributes to the development and metastasis of multiple cancer types 4, 5. The Wnt signaling pathway is commonly divided into either β-catenin-dependent (canonical) or -independent (non-canonical) signaling. Wnt proteins are secreted ligands that regulate numerous developmental processes. In canonical Wnt signaling, Wnt binds to members of Frizzled family and LRP5/6, activating cytoplasmic β-catenin which in turn translocates to the nucleus and targets members of the LEF/TCF transcription factor family to activate downstream effector genes such as *c-MYC, CCND1* and *MMP7*. This gene expression regulation precisely orchestrates cell fate and morphogenesis 6. In non-canonical Wnt signaling, Wnt regulates planar cell polarity by stimulating cytoskeletal reorganization and calcium mobilization, or through RhoA/JNK signaling 7.

The R-spondins are a family of four secretory ligands. Encoded by *RSPO1-4*, R-spondins were initially found to act as Wnt agonists or potentiators which play important roles in embryonic development and adult stem cell maintenance 8. Recently, R-spondin family members have been found to promote or suppress cancer progression by regulating different signaling pathways including canonical and non-canonical Wnt signaling 9, 10. For example, RSPO1 suppresses metastasis of colon cancer through activating TGFβ signaling 11. RSPO2 promotes the stemness of susceptible pancreatic tumor cells through enhancing canonical Wnt signaling 12 and the self-renewal of acute myeloid leukemia cells via inhibiting BMP signaling 13. RSPO2 also drives liver tumor development in a Yes-associated protein (YAP)-dependent manner 14 and promotes the development and metastasis of squamous cell carcinoma of the tongue through canonical Wnt signaling 15. Meanwhile, RSPO2 exhibits an inhibitory effect on cancer development by antagonizing canonical Wnt signaling 16 and metastasis by counteracting Wnt5a/Fzd7-driven non-canonical Wnt pathway 17. RSPO3 drives the development of CRC through canonical Wnt signaling 18 and enhances the aggressiveness of Keap1-deficient lung adenocarcinoma (LUAD) through interacting with LGR4 19. RSPO4 is reported to be involved in nail development, liver fibrogenesis and periodontitis 20-22. Interestingly, RSPO4 is unique among the R-spondin family. Different R-spondins show considerable differences in their binding affinities towards ZNRF3/RNF43 with RSPO4 having the lowest affinity. Concordantly, RSPO4 also has the lowest efficacy in potentiating Wnt/β-catenin signaling. RSPO4 drug-conjugates targeting LGR4/5/6 simultaneously generated robust anti-tumor effect 23, 24, implying its tumor suppressive roles in human cancers. Thus, the functional effects of R-spondins on tumor cells vary in different cancerous context.

The expression of RSPO family members is regulated by different genetic and/or epigenetic mechanisms in cancerous context. For example, overexpression of *RSPO2* and *RSPO3* caused by chromosomal rearrangement drives Wnt-dependent development of colon cancer 25, 26. Copy number amplifications of *RSPO2* and *RSPO3* are associated with poor prognosis in breast cancer 27. Inactivation of *RSPO1* by promoter CpG methylation was found in acute lymphocytic leukemia 28, and *RSPO2* methylation frequently occurred in CRC 16. *RSPO3* methylation was found to promote the progression of cholangiocarcinoma 29. *RSPO4* overexpression was reported in breast cancer, while its regulatory mechanism remains unknown 30. Recently, it was found that *RSPO4* expression was upregulated by the lncRNA UNC5B-AS1/miR-4455 axis in cervical cancer 31. Therefore, the expression of RSPOs were regulated by different mechanisms in cancer cells.

R-spondin family members contain two furin-like cysteine-rich domains (FU1 and FU2), followed by a thrombospondin domain (TSP) and a C-terminal basic region (BR) with variable length 32. In the absence of R-spondins, RNF43/ZNRF3 function as E3 ubiquitin ligase to degrade Wnt receptor Fzd/LRP5/6 complexes at the cell membrane 8. When present, R-spondins recruit LGR4/LGR5 through its FU2 domain, and then binds RNF43/ZNRF3 through its FU1 domain, resulting in the internalization and membrane clearance of the R-spondins-LGR4/5-RNF43/ZNRF3 complex. Consequently, without the degradation by RNF43/ZNRF3, Wnt/Fzd/LRP5/6 receptor complexes stay on the plasma membrane, thus enhancing Wnt activity 8. In this scenario, R-spondins function as Wnt agonists. Some R-spondins, however, might function as Wnt antagonists in certain contexts. For example, RSPO2 inhibits the development of colon cancer through suppressing Wnt/β-catenin signaling. In this scenario, RSPO2 binds to LGR5, but not LGR4, and stabilizes ZNRF3. ZNRF3 degrades Wnt receptors and blocks Wnt/β-catenin signaling, which in turn upregulates the Wnt target gene LGR5 16. Here, RSPO2 inhibits Wnt/β-catenin signaling in this context by forming a negative feedback loop. The RSPO1-LGR5 axis can exhibit a similar effect in HEK293 cells 33. Thus, differential tissue- and context-dependent activities and mechanisms of the four R-spondins have been reported with respect to their regulation of Wnt/β-catenin signaling.

Epithelial-mesenchymal transition (EMT) is a process of reversible cellular alterations during which epithelial cells progressively lose their cobblestone epithelial appearance in monolayer cultures to adopt a spindle-shaped, mesenchymal morphology 34. Cells may individually or collectively acquire mesenchymal features and increase motility and invasive abilities. EMT is involved in most steps of cancer cell invasion and metastasis 35. Wnt/β-catenin signaling directly regulates EMT through targeting and activating EMT transcription factors such as *SNAI* which regulate the expression of E-cadherin and N-cadherin, or through adherens junctions by other Wnt/β-catenin-targets such as *MMP7* 34. The activation of EMT results in quasi-mesenchymal cells which display front-rear polarity and extensively reorganizes the actin stress fibers that form an important component of their cytoskeleton 36. A tumor-initiating or cancer stem cell (CSC) state can be observed during the EMT process 37. Induction of EMT in epithelial cells will cause the expression of stem cell markers and enable *in vitro* tumor sphere-forming capabilities 38. The RSPO/LGR5 axis has been found to play important roles in stem cell formation of different origins, including colon 39, kidney 40, liver 41, stomach 42, pancreas 43 and gallbladder 44. However, the role of the RSPO/LGR5 axis in CSCs remains largely unknown.

We previously explored methylated TSG candidates for multiple types of human cancers through CpG methylomic study 45, 46, and identified *RSPO4* as a methylated target. In this study, we systematically evaluated *RSPO4* expression status in a variety of cancers, and further evaluated its functions and underlying mechanisms in cancer stemness and metastasis.

## Materials and Methods

### Database mining

Databases including cBio (MSKCC) 47 and Catalogue of Somatic Mutations in Cancer (COSMIC) (Wellcome-Sanger) 48 were screened for information specifying genomic alterations, DNA methylation and mRNA expression in TCGA cohorts, cell lines and other published papers. DNA methylation and mRNA expression data in cancer tissues and normal controls was retrieved from DNMIVD (DNA methylation interactive visualization database) 49. DNA methylation data was also retrieved from UALCAN 50. The mRNA expression data in cancer tissues and normal controls was retrieved from SangerBox 51. MethSurv was used to analyze the correlation between methylation levels and overall survival for pan-cancer patients 52. Kaplan-Meier survival analysis was plotted by KM-plotter and PrognoScan with Log-rank p-value calculated 53, 54. The damaging effect of *RSPO4* mutation was evaluated by PolyPhen-2 software (Version 2.2.2) 55.

### Cell lines and tissue samples

A series of tumor cell lines were used in this study, including glioma, NPC, ESCC, Lung Ca, CRC and Blad Ca cell lines 56. Immortalized cell lines (NP69, Het-1A, NE1 and NE3) were used as normal control with culture conditions 57. HCT116 *DNMT1*-/- *DNMT3B*-/- (DKO) cells were kind grift from Dr. Bert Vogelstein (Johns Hopkins University) and were grown with either 0.4 mg/ml genecitin or 0.05 mg/ml hygromycin 58. All tumor cell lines were cultured in RPMI 1640 (Invitrogen) with 10% FBS and routinely evaluated for mycoplasma contamination. Cell lines used in this study were purchased from either American Type Culture Collection (ATCC) or from collaborators. Human normal adult and fetal tissue RNA were purchased commercially (Stratagene, La Jolla, CA, USA; Millipore-Chemicon, Billerica, MA). DNA samples of normal, primary carcinomas and matched surgical margin tissues have been described previously 59.

### Semi-quantitative RT-PCR, bisulfite treatment and promoter methylation analysis

RT-PCR, bisulfite treatment of DNA, methylation-specific PCR (MSP) and bisulfite genomic sequencing (BGS) were performed as previously described 59. Primers used for RT-PCR, MSP and BGS were listed in Table S1. RT-PCR primers for genes regulated by *RSPO4* expression were listed in Table S2.

### Demethylation treatment

Treatment of tumor cell line using Aza and TSA was performed to restore *RSPO4* expression. Cells were treated with 10 uM Aza (Sigma, Ronkonkoma, NY) for 72 h. After 72 h of Aza treatment, cells were treated with 100 ng/ml of TSA for additional 24 h and harvested for DNA and RNA preparation.

### Plasmid construction and generation of stable cell line

The full-length Open Reading Frame (ORF) of *RSPO4* was cloned to pcDNA3.1 (+) vector, with a V5 tag to C terminal of *RSPO4*. Also, we generated mutants inactivating each domain of RSPO4 in the plasmid pcDNA3.1 (+)-Flag-*RSPO4*-V5 using PCR site-directed mutagenesis methodology. All the clone primers are listed in Table S3. *LGR4* and *LGR5* expression plasmids were kindly gifted from Dr. Qingyun Liu (the University of Texas-Houston Health Science Center) 33. *ZNRF3* expression plasmid was kindly gifted from Dr. Feng Cong (Novartis Institutes for Biomedical Research) 60.

We used the T-REx^TM^ system (Invitrogen) to generate stable cell line. The inducible expression plasmid pcDNA3.4-*RSPO4*-V5 was linearized and then transfected into the HNE1 cell line using Lipofectamine 2000 (Invitrogen) with blasticidin (5 ug/ml) and genecitin selection (400 ug/ml; Calbiochem, Darmstadt, Germany) for 3~4 weeks. The resistant clones were confirmed by Western blot. One colony was selected, and the cells were incubated with tetracycline (Tet, 1 ug/ml) induction for 24 hrs and then collected for further analyzed.

### RNA interference

Small interfering RNA (siRNA, OriGene Technologies, Rockville, MD) was used to knockdown *RSPO4*, *LGR4* and *LGR5* expression in cancer cells. For siRNA transfections, 1~2×10^5^ cells were seeded in 6-well plates and transfected with 30 pmol siRNA using 2 ul Lipofectamine 2000 (Invitrogen). 48 hrs later, cells were harvested for analysis. For dual-luciferase reporter assay, 5000 cells were seeded in 96-well culture plates, 5 pmol siRNA were transfected into cells using 0.25 ul Lipofectamine per well.

### Conditioned medium

Conditioned media (CM) were generated to detect the secretion of RSPO4 and its mutant protein. A549 and KYSE150 were seeded into 6-well culture plates, when 70 ~ 80% confluences, empty vector, *RSPO4* and FUm1/2 mutant were transiently transfected by using Lipofectamine 2000 (Invitrogen) with serum-free RPMI1640 medium.

RSPO4 and FUm1/2 mutant protein was extracted from conditional medium using trichloroacetic acid (TCA, Sigma). 48 hrs after transfection, the medium was collected. TCA was added into the medium overnight at -20 °C then centrifuged at 16,100 g for 45 min at 4 °C to collect precipitated proteins. Then the collected protein pellets were dissolved in 4×Laemmli sample buffer for further analysis.

### Monolayer and soft agar colony assays

Colony formation assay was performed to evaluate cancer cell growth and proliferation. After 48 hrs transfection of *RSPO4* and FUm1/2 mutant, or si-*RSPO4* and control, the transfected cells were subcultured into 6-well plates for genecitin selection. After 8~14 days of selection, surviving colonies (> 50 cells per colony) were stained with gentian violet and counted. The experiments were performed three times in technical triplicate.

Anchorage-independent growth of tumor cells was determined by soft agar assay. The transfected tumor cells were suspended in full RPMI1640 medium containing 0.35% agar and 400 µg/ml of genecitin in 12-well plates. Colonies were photographed and counted after about 2 weeks of selection. The experiments were performed three times in technical triplicate.

### *In vivo* xenograft models

Female BALB/c nude mice aged 4 weeks were used for tumor implantation experiments. Empty vector or *RSPO4*-expressing LoVo cells (2-5 × 10^6^ cells in PBS) were injected subcutaneously into the flanks of nude mice with randomization (n = 10). No blinding to the group allocation during the experiment was done. Starting on day 10 after the first injection, tumor growth was monitored once every 7-10 days for 40 days according to the actual tumor formation and animal welfare ethics regulations (tumor diameter < 20 mm). Tumor volume was calculated as [π/6 × L (length) × W (width) × H (height)]. All animal work was approved by the Institutional Ethics Committees of the Chinese University of Hong Kong.

### Flow cytometry analysis

Flow cytometry analysis was performed to analyze cell cycle and apoptosis. Cancer cells were used for detecting the effect of *RSPO4* expression after 48 hrs transfection of vector and *RSPO4*. Cells transiently transfected with control and *RSPO4* siRNA were used for testing its effect of *RSPO4* depletion. All cells were collected for analysis after 48 hrs transfection. For cell cycle analysis, cells were fixed in ice-cold 70% ethanol and stained with PI. For apoptosis analysis, tumor cells were collected after 48 hours transfection and then stained with Annexin V-FITC and PI by using FITC Annexin V Apoptosis Detection Kit II following the manufacturer's instructions (#556570, BD Pharmingen™). The Annexin V-positive cells were counted as apoptotic cells. Cell-cycle and apoptosis profiles were obtained using the C6 Flow Cytometer® Instrument (BD Biosciences, San Jose, CA, USA) with cell cycle data analyzed by ModFit LT™ Highlights software and apoptosis by BD Accuri C6 Software. The experiments were performed three times.

### Scratched wound-healing, migration and invasion assays

Wound healing assay was performed to evaluate cell motility. For the migration assay, transfected cells (2.5×10^4^ per well) were seeded into a Transwell plate (Corning, NY). For the invasion assay, 2.5×10^4^ transfected cells were plated in each well of a Corning BioCoat Matrigel Invasion Chamber (Corning, NY). In the upper insert, cells were suspended in serum-free RPMI1640 medium, with 5% FBS medium in the lower chamber as chemoattractant. After 18~24 hours of incubation, migrated or invaded cells were fixed and stained. Different fields of cells were photographed, and numbers of cells were counted. The experiments were performed three times.

### Sphere formation assay

After vector, *RSPO4* and FUm1/2 transfection, cancer cells were cultured at a density of 5,000 cells/well in 24-well ultralow attachment plates (Corning, Corning, NY) at 37ºC in serum-free DMEM/Nutrient Mixture F-12 (DMEM/F12) (1:1) (Gibco Life Science, Great Island, NY), supplemented with 1% penicillin/streptomycin, 1 × B27 (Gibco Life Science), 4 mM HEPES (Sigma-Aldrich, St. Louis, MO), 20 ng/ml basic fibroblast growth factor (PeproTec, Rocky Hill, NJ), 20 ng/ml EGF (PeproTec), and 1 × insulin-transferrin-sodium selenite (Sigma- Aldrich). Growth factor-enriched conditions were maintained by adding supplements every 2 days. The total number and size of spheres were analyzed on day 7. Images of the spheres were obtained using an Olympus BX51 microscope (Olympus Corporation, Tokyo, Japan).

### Dual-luciferase reporter assay

TOPflash/FOPflash, *c-MYC*, *CCND1* and *MMP7* luciferase activities were analyzed by using dual luciferase reporter assays. Luciferase reporter was co-transfected with *RSPO4* (or *si-RSPO4#A* or* #C*; *siLGR4* and *siLGR5*) or empty vector (or si-Control), together with an internal control *Renilla reniformis* luciferase reporter pRL-CMV vector by using Lipofectamine 2000 (Invitrogen). Forty-eight hours after transfection, cells were harvested and analyzed by Dual-Luciferase Assay Kit (Promega, Madison, WI). The experiment was conducted three times in technical triplicate.

### Immunoblotting and immunoprecipitation

Human RSPO4 recombinant protein was commercially purchased (R&D Systems #4575-RS/CF). Total cell lysates were prepared by lysing cells using RIPA buffer (50mM Tris-HCl, pH 7.4, 150mM NaCl, 1% NP-40, 0.25% sodium deoxycholate and 1mM EDTA) supplemented with protease inhibitors and phosphatase inhibitors, followed by centrifugation at 14,000 rpm for 10 min at 4 °C. Equal amount of proteins were resolved by SDS-PAGE, transferred to nitrocellulose membranes. The membranes were incubated with primary antibody at 4°C overnight, followed by incubation with secondary antibody at room temperature for 45 min. Immunoreactive bands were detected by Western blot luminol reagent (GE Healthcare, Waukesha, WI). Co-immunoprecipitation experiments were performed as described previously 60. Briefly, membrane proteins were extracted by using Mem-PER^TM^ Plus Membrane Protein Extraction Kit (Thermo Scientific #89842) following the manufacturer's instructions. Membrane fractions were incubated with the V5 antibody and Protein G-sepharose beads (Amersham) overnight at 4°C. Beads were washed four times with lysis buffer and the bound proteins were eluted in 4×Laemmli sample buffer for immunoblotting analysis.

Antibodies used included: V5 (#MCA1360GA, AbD Serotec); cleaved caspase-3 (#9661), cleaved caspase-7 (#9491), cleaved caspase-9 (Asp330) (#9501), cleaved poly (ADP-ribose) polymerase (#9541), LRP6 (#2560), phospho-LRP6 (Ser1490) (#2568), phospho-β-catenin (Ser552) (#9566), phospho-AKT (Ser473) (#4060), AKT (#4691); p-SAPK/JNK (#9251S), Phospho-p44/42 MAPK (Erk1/2) (Thr202/Tyr204) (#9101), p44/42 MAPK (Erk1/2) (#9102), RhoA (67B9) (#2117), Src (#2108), phospho-Src Family (Tyr416) (D49G4) (#6943), phospho-c-Jun (Ser63) II (#9261) and E-cadherin (#3195) (Cell Signaling, Beverly, MA); phospho-RhoA (Ser188) (#PA5-105763); Vimentin (#V6630, Sigma-Aldrich) and active β-catenin (#05-665, Upstate, Lake Placid, NY, USA); total β-catenin (#M3539), anti-mouse IgG-HRP (#P0161), anti-rabbit IgG-HRP (#P0448) (Dako, Glostrup, Denmark); N-cadherin (BD Transduction Labs, San Jose, CA, USA); Fibronectin (#sc-9068), phospho-RhoA (Ser188) (#sc-32954), c-MYC (#sc-764), LGR4 (#sc-390630) (Santa Cruz Biotechnology, TX, USA); ZNRF3 (#R2407-vp) (Abiocode, CA, USA); LGR5 (#PA5-35304), MMP7 (#MS-813-P0) (ThermoFisher Scientific); Cyclin D1 (#M7155, Dako); α-Tubulin (Lab Vision Corporation, Fremont, CA, USA); anti-RSPO4 (Atlas Antibodies Cat# HPA048887, RRID: AB_2680545).

### Indirect immunofluorescence

Cells grown on coverslips were stained by indirect immunofluorescence. Cells were incubated with primary antibody against V5, E-cadherin or Vimentin at 37 °C for 30min or 4 °C overnight, and then incubated with Alexa Fluor 594 or 488- (Invitrogen Molecular Probes, Carlsbad, CA, USA) conjugated secondary antibody against mouse or rabbit IgG at 37 °C for 30min. Cells were then counterstained with DAPI and imaged with an Olympus BX51 microscope (Olympus Corporation, Tokyo, Japan).

For stress fiber formation assay, cells were cultured in serum free medium for 24h before serum induction. Then stress fiber formation was induced by incubation in normal medium with 10% FBS for 2 h before the fixation of transfected cells and immunofluorescence staining. Cells were fixed and stained by Rhodamine-labeled phalloidin (Invitrogen Molecular Probes). Cells were then counterstained with DAPI and imaged with an Olympus BX51 microscope (Olympus Corporation, Tokyo, Japan).

### Statistical analysis

The Student's t-test or one-way ANOVA was performed to determine whether differences between the experimental and control groups were significant. Results were displayed as values of mean ± standard deviation (SD). For all tests, the criteria for significance were *p* < 0.05 (*), *p* < 0.01 (**), and *p* < 0.001 (***) for all comparisons. Pearson's χ^2^ test was used for comparison of patient characteristics and methylation status.

## Results

### Identification of *RSPO4* as a methylated target with clinical significance

**Figure 1:**
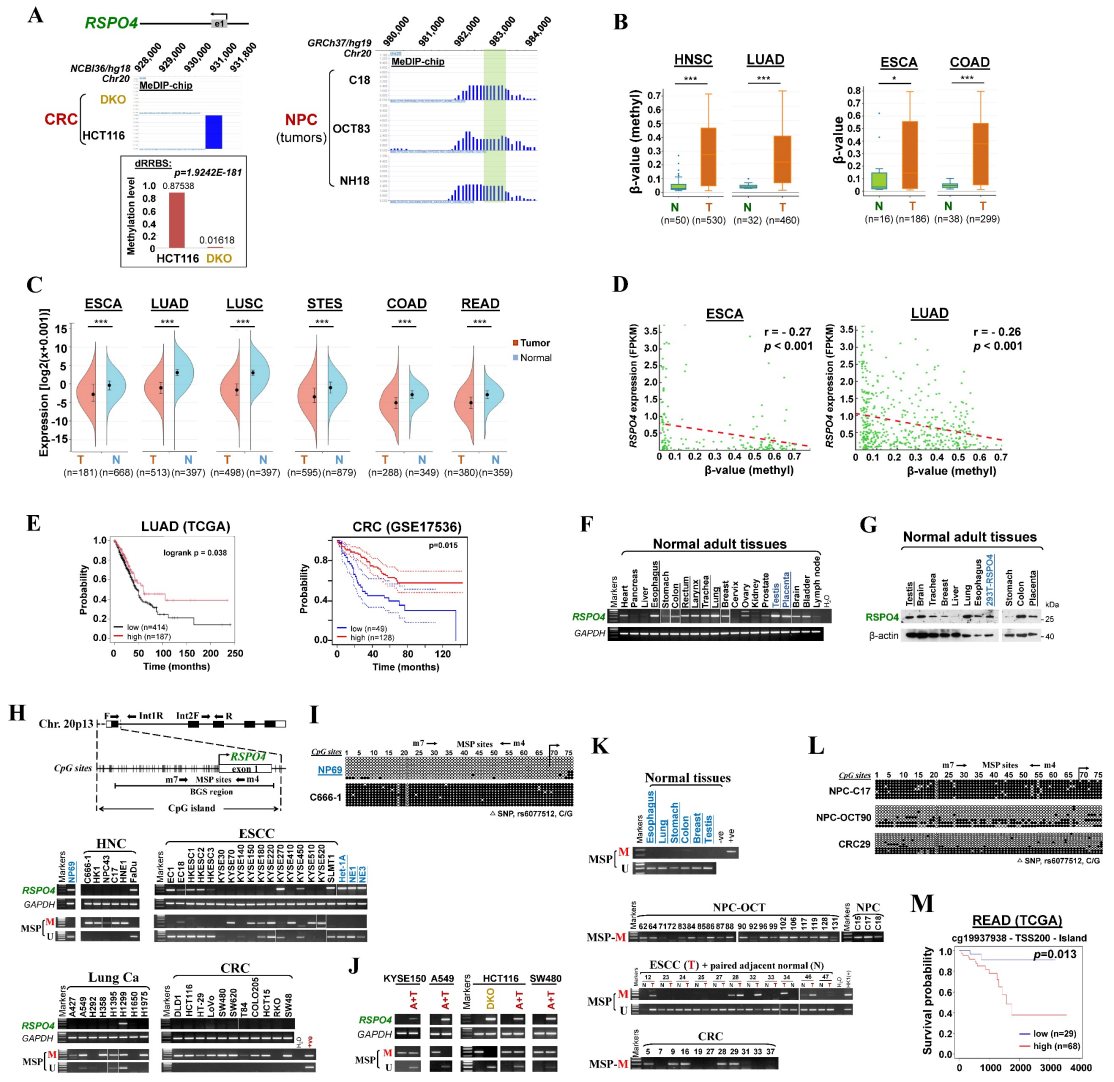
** Identification of *RSPO4* as a methylated target gene with clinical significance.** (A) MeDIP-chip study identified *RSPO4* as a methylated target in CRC and NPC cell lines and primary tumors (C18, OCT83 and NH18). *RSPO4* gene structure, promoter and exon 1 (UCSC Genome Browser NCBI36/hg18) are shown on the top panel. e1: exon 1. Positive methylation signal peak (blue) in HCT116 was identified by methylated DNA immunoprecipitation (MeDIP)-chip. Promoter CpG methylation of *RSPO4* was also identified by double-enzyme reduced representation bisulfite sequencing (dRRBS) in HCT116 and its double knock-out of *DNMT1* and *DNMT3A* (DKO) cells (bottom panel). (B) β-value as the indicator of methylation level of *RSPO4* in cancer tissues and the normal control in TCGA datasets, as retrieved from DNMIVD. HNSC, head and neck squamous carcinoma; LUAD, lung adenocarcinoma; ESCA, esophageal carcinoma; COAD, colon adenocarcinoma; N, normal control; T, tumor. (C) *RSPO4* mRNA expression levels in different cancer types in TCGA datasets, as retrieved from SangerBox. LUSC, lung squamous cell carcinoma; STES, stomach and esophageal carcinoma; READ, rectum adenocarcinoma. (D) Analyses of TCGA datasets reveal an inverse correlation between mRNA expression level and promoter methylation level of *RSPO4* in ESCA and LUAD, as retrieved from DNMIVD. Each green circle represents a single clinical sample. Pearson correlation coefficient analysis is used. (E) Kaplan-Meier curve analyses show the association between *RSPO4* mRNA expression and overall survival of patients with LUAD in TCGA datasets, as retrieved from KM-plotter, and CRC as retrieved from PrognoScan. CRC, colorectal carcinoma. (F) RT-PCR detected *RSPO4* mRNA expression in a panel of human normal adult and fetal tissues. (G) Western blot detected *RSPO4* protein level in a panel of human normal adult and fetal tissues. 293T cell line ectopically expressing *RSPO4* was used as a positive control. (H) Schematic structure of *RSPO4* promoter. The primers for RT-PCR and multiplex DNA PCR are indicated with arrows. Exon 1, CpG sites (short vertical lines), MSP sites and BGS region analyzed are shown. RT-PCR and MSP detected *RSPO4* mRNA expression and promoter CpG methylation in cancer cell lines and non-transformed epithelial cell lines (NP69, Het-1A, NE1 and NE3), respectively. M, methylated; U, unmethylated. Ca, carcinoma; NPC, nasopharyngeal carcinoma; ESCC, esophageal squamous cell carcinoma. (I) BGS analysis of *RSPO4* promoter in non-transformed epithelial cell line (NP69) and cancer cell line (C666-1). Each row of circles represented an individual promoter allele. Filled circle, methylated CpG site; open circle, unmethylated CpG site; open triangle, SNP rs6077512 (C/G). (J) *RSPO4* mRNA expression in methylated/silenced cancer cell lines was detected by RT-PCR after pharmacologic demethylation treatment with Aza combined with TSA (A+T). (K) MSP analysis of *RSPO4* methylation in different types of primary tumor tissues. Representative samples are shown. (L) BGS analysis of *RSPO4* methylation pattern in representative primary tumor tissues and normal tissues. (M) Kaplan Meier analysis shows the association between *RSPO4* promoter methylation at specific CpG sites and overall survival of patient with READ from TCGA datasets, as retrieved from MethSurv. The methylation patient groups are dichotomized by higher (β > cut-off) and lower (β < cut-off), according to a best cut-off point in MethSurv.

Through analyzing whole-genome CpG methylation profiles (methylomes) by methylated DNA immunoprecipitation (MeDIP)-chip and double-enzyme reduced representation bisulfite sequencing (dRRBS) 45, we identified *RSPO4* as a methylated gene in cancer cell lines as well as tissues (Fig. 1A). Through analyzing TCGA datasets using DNMVID 49, we found that *RSPO4* exhibited significant higher methylation level in multiple types of cancer tissues than their corresponding normal tissues (Fig. 1B). By analyzing TCGA datasets using Sangerbox 51, we found that mRNA expression of *RSPO4* was significantly lower in multiple cancer tissues than the corresponding normal tissues (Fig. 1C). Further analysis using DNMVID indicated that *RSPO4* expression was negatively correlated with its methylation level (Fig. 1D). These results indicated that *RSPO4* expression is likely regulated by CpG methylation and its silence/downregulation occurs frequently in multiple types of carcinomas.

In addition, by analyzing the overall survival using KM-plotter 53 and PrognoScan 54, we found that lower *RSPO4* expression was significantly associated with worse overall survival in patients with different types of cancer (Fig. 1E). We also analyzed the association between *RSPO4* methylation and clinical features in cancer patients from TCGA datasets. The χ^2^ analysis revealed a significant association between *RSPO4* methylation and diagnosis age (*p* < 0.01), histological type (*p* < 0.001) and tumor size (*p* < 0.05) in CRC patients (Table 1). We also found a significant association between *RSPO4* methylation and diagnosis age (*p* < 0.001), neoplasm histologic grade (*p* < 0.05), Karnofsky performance score (*p* < 0.001), ethnicity (*p* < 0.005) and asthma history (*p* < 0.05) in patients with brain lower grade glioma (Table S4). Therefore, *RSPO4* methylation is associated with poor prognosis and clinicopathological features of cancer patients.

### *RSPO4* promoter methylation is frequently detected in tumor cell lines and primary carcinomas

To verify findings from our CpG methylomic study and public database analysis, we examined the expression and methylation of *RSPO4* in normal tissues, cancer cell lines and primary tumor tissues. Semi-quantitative reverse transcription (RT)-PCR data showed that *RSPO4* was readily expressed in most normal human adult and fetal tissues (Fig. 1F). Western blot also detected the endogenous expression of RSPO4 protein in a panel of human normal tissues, at the same size with ectopically expressed RSPO4 protein (Fig. 1G). Further RT-PCR analysis showed that *RSPO4* was well expressed in immortalized normal cell lines (Fig. 1H), but frequently silenced or downregulated in a variety of carcinoma cell lines including nasopharyngeal cancer (NPC), esophageal squamous cell carcinoma (ESCC), lung cancer (Lung Ca), colorectal cancer (CRC), bladder cancer (Blad Ca) and ovary cancer (OvCa) (Fig. 1H and Fig. S1A).

We further analyzed the *RSPO4* promoter and found that it contained a typical CpG island (Fig. 1H), suggesting that *RSPO4* is susceptible to CpG methylation-mediated silencing. We then assessed *RSPO4* promoter methylation using methylation-specific PCR (MSP) and found that *RSPO4* promoter was frequently methylated in cell lines of NPC, ESCC, Lung Ca, CRC, Blad Ca and OvCa, well correlated with its expression levels (Fig. 1H and Fig. S1A, Table 2). Bisulfite genomic sequencing (BGS) detected high density of methylated CpG sites within the region spanning core promoter and exon 1 of *RSPO4* in representative tumor cell lines (Fig. 1I and Fig. S1B). In contrast, methylation was not observed in normal epithelial cell lines (Fig. 1H and 1I, Fig. S1B), suggesting that *RSPO4* methylation is cancer-specific and common in multiple cancer cell lines. We also noticed that methylation was not detected in several cell lines with downregulated *RSPO4* (Fig. 1H), indicating that other regulatory mechanisms such as genomic deletions might also be involved. By using multiplex DNA PCR, we confirmed that genomic deletion occurred in these cell lines (A427, H292, T84, RKO and some KYSE cell lines) (Fig. S1C).

To further confirm whether methylation directly contributed to *RSPO4* silencing, cancer cell lines with *RSPO4* methylation/silencing were treated with DNA methyltransferase inhibitor 5-aza-2'-deoxycytidine (Aza) in conjunction with HDAC inhibitor trichostatin A (TSA). MSP analysis suggested that Aza plus TSA treatment successfully restored *RSPO4* expression in these cell lines, accompanied by the appearance of unmethylated alleles (Fig. 1J). Complete demethylation of the *RSPO4* promoter and full restoration of its expression was also seen in colon cell line HCT116 with genetic double knock-out of DNMT1 and DNMT3B (DKO) (Fig. 1J), indicating that the CpG methylation of *RSPO4* promoter is controlled by DNMT1 and DNMT3B. Demethylation of *RSPO4* promoter in cancer cell lines was also confirmed by BGS analysis (Fig. S1B). These results demonstrate that aberrant promoter CpG methylation mediated the transcriptional silencing/downregulation of *RSPO4* in multiple carcinomas.

We further examined *RSPO4* methylation in primary tumor samples. MSP analysis showed that *RSPO4* was methylated in 96% (22/23) of NPC, 45% (5/11) of CRC and 39% (24/62) of ESCC samples (Fig. 1K, Table 2). BGS analysis confirmed the methylation in representative tumor samples (Fig. 1L). Through analyzing the TCGA datasets, we found that *RSPO4* was frequently methylated in a variety of TCGA cancer cohorts, with genomic deletions also exist occasionally (Table 3, Fig. S1D). By mining mutation data from COSMIC and TCGA, we found that *RSPO4* also underwent truncating and homozygous point mutations, indicating its loss-of-function effect in cancers and genetic diseases (Table 3, Fig. S1D, Table S5 and S6). Collectively, based on its frequent silencing/downregulation, loss-of-function mutations, and copy number loss in multiple cancer types, we conclude that *RSPO4* is likely a tumor suppressor in human cancers. Methylation analysis using MethSurv also indicated that higher *RSPO4* methylation level with specific probes was associated with worse overall survival of patients with rectum adenocarcinoma (READ) (Fig. 1M).

### *RSPO4* encodes a secreted protein which inhibits tumor cell proliferation

**Figure 2:**
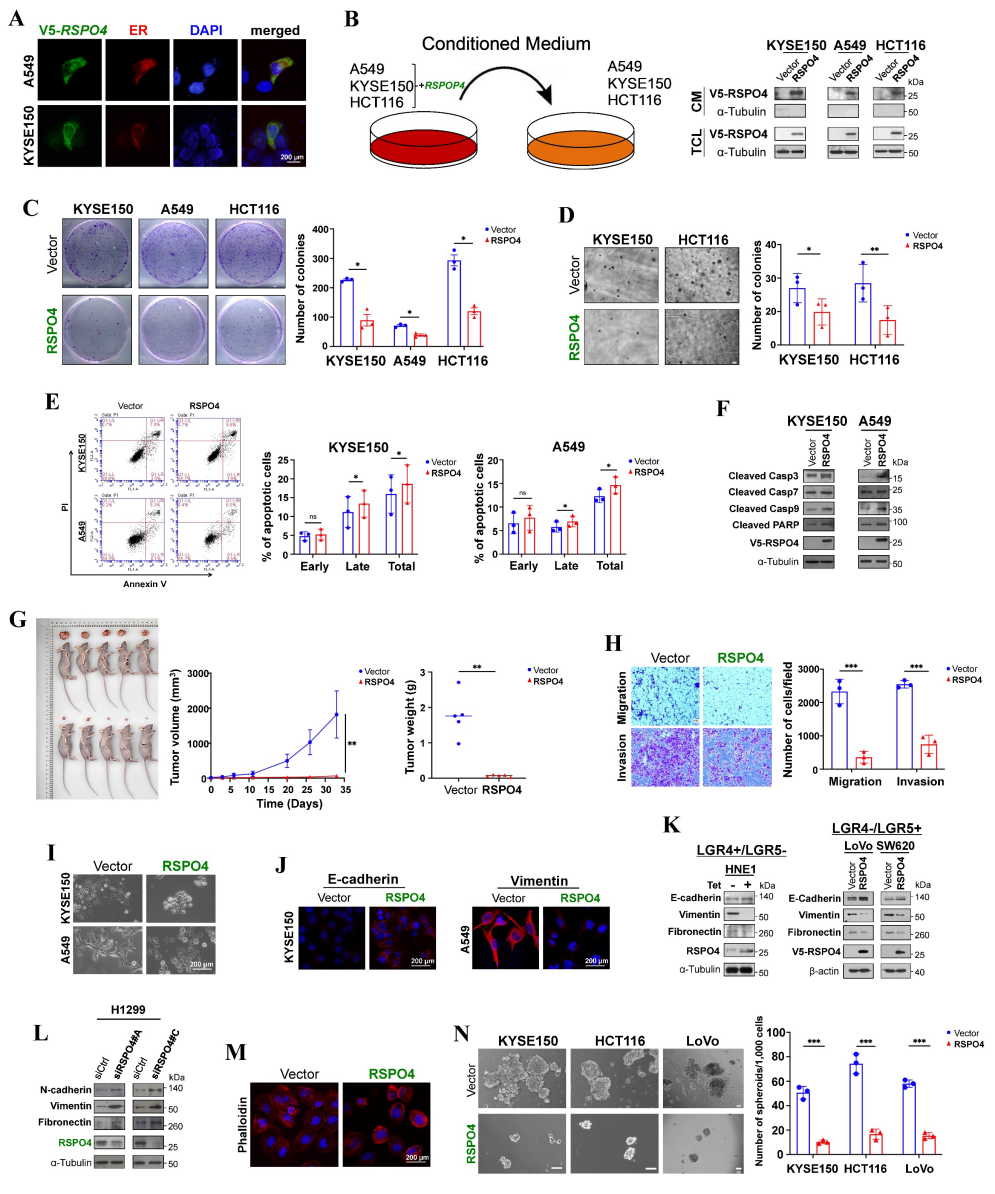
***RSPO4* encodes a secreted protein which inhibits tumor cell clonogenicity, migration, invasion and stemness.** (A) Subcellular localization by immunofluorescence showed that RSPO4 protein co-localized with the endoplasmic reticulum (ER). Original magnification, ×400. Scale bar, 200μm. (B) RSPO4 protein can be detected in conditioned medium after 24 hrs posttransfection. CM, serum-free conditioned medium; TCL, total cell lysates. (C) Monolayer CFA in KYSE150, A549 and HCT116 cells. 5,000 cells were seeded in each well and colonies were counted after 2 weeks. (D) Anchorage-independent soft agar assay on KYSE150 and HCT116 cells. 5,000 cells were seeded in each well and colonies were counted after 4 weeks. (E) Flow cytometry analysis of apoptosis by Annexin V-FITC/PI staining of A549 and KYSE150 cells. Both early and late apoptotic cells (Annexin V-positive) were counted. (F) Western blot detected the protein level of cleaved caspase 3, 7, 9 and PARP in cancer cells transfected with vector- and *RSPO4*. (G) *In vivo* tumor formation ability of LoVo cells transduced with lentivirus encoding *RSPO4* or empty vector, then injected subcutaneously into BALB/c nude mice. (H) Transwell migration and invasion assay of HONE1 cells transfected with empty vector and *RSPO4* plasmid. (I) Morphological changes in *RSPO4*-transfected cancer cells compared with vector control after genecitin selection for 2 weeks. Original magnification, ×400. Scale bar, 200μm. (J) Indirect immunofluorescence staining of E-cadherin and vimentin in empty vector- and *RSPO4*-transfected KYSE150 and A549 cells, respectively. Original magnification, ×400. Scale bar, 200μm. (K) Western blot detected the expression levels of E-cadherin, vimentin and fibronectin in *RSPO4*-stably (*Left*) and transiently expressed cancer cells (*Right*). (L) Western blot detected the protein level of vimentin, N-cadherin and fibronectin in H1299 cells with knockdown of *RSPO4* by siRNAs. (M) Effect of ectopic RSPO4 expression on cytoskeletal structures of A549 cells. Red, Rhodamine-labeled phalloidin; Blue, DAPI. Original magnification, ×400. Scale bar, 20μm. (N) Sphere-forming assays evaluated the stemness of cancer cells transfected with empty vector and *RSPO4* plasmid. Scale bar, 100 μm. For C, D, E, F, H and N, n = 3 biologically independent replicates were examined over three independent experiments with similar results. Data are presented as mean values ± SD. For C, D, E, H and N, Student's test was performed to obtain the P values. For G, n=5 mice were used for *RSPO4* and vector control, and one-way ANOVA was performed to obtain the P values.

R-spondins have been identified as secreted proteins. R-spondin 4 has a putative signal peptide and shares high homology with other R-spondin family members (Fig. S2A). We transfected expression plasmids encoding V5-tagged *RSPO4* into A549 and KYSE150 tumor cells and examined its subcellular localization by indirect immunofluorescence. RSPO4 protein was detected mainly in endoplasmic reticulum (ER) (Fig. 2A). In similarly transfected cells, high levels of RSPO4 protein in the culture media (CM) were detected by Western blot (Fig. 2B). These results confirmed that *RSPO4* encoded a secretory protein like other R-spondin family members.

We then explored the function of *RSPO4* by evaluating its effect on tumor cell growth. We selected cell lines with complete methylation and silencing status. Colony formation assay (CFA) showed that the numbers of colonies were significantly less in tumor cells with ectopic expression of *RSPO4* than the vector control (Fig. 2C), and more colonies were observed with knockdown of *RSPO4* expression (Fig. S2B). Anchorage-independent soft agar assay showed that colony numbers were significantly decreased in tumor cells with *RSPO4* expression, along with reduced colony size, compared with vector controls (Fig. 2D).

To decipher the underlying mechanisms of *RSPO4*-mediated inhibition of tumor cell growth, we checked *RSPO4* effect on the cell cycle and apoptosis of tumor cells using flow cytometry after propidium iodide (PI) and Annexin-V-FITC/PI dual staining. In both tumor cell lines, when *RSPO4* was expressed, a significant increase in cells in S phase was observed, with corresponding decrease in cells in G0/G1 phase (*p* < 0.05) (Fig. S2C); the opposite was observed in tumor cells with *RSPO4* knockdown (Fig. S2D). As *RSPO4* expression inhibits tumor cell proliferation, the slight increase in S phase cells could be explained by the accumulation of cells arrested in this phase, rather than cells actively replicating DNA.

We also performed flow cytometry to check whether *RSPO4* expression can induce cancer cell apoptosis. We found that *RSPO4* expression induced increased apoptosis in both A549 and KYSE150 (*p*<0.05) cells compared with the empty vector (Fig. 2E). To further confirm the effects of *RSPO4* on apoptosis, key mediators of apoptosis were examined by Western blot. We observed that ectopic *RSPO4* expression upregulated apoptosis markers involved in intrinsic apoptotic pathway, including cleaved caspase 3, caspase 7, caspase 9 (Asp330) and poly (ADP-ribose) polymerase (PARP) (Fig. 2F). These results indicated that RSPO4 suppressed tumor cell growth through inducing S-phase arrest and intrinsic apoptotic pathway.

A tumor xenograft model was used to investigate whether *RSPO4* expression could suppress tumor formation *in vivo*. LoVo cells with stably expressed *RSPO4* or control vector were injected into nude mice, with tumor formation efficiency monitored across different time points. Ectopic *RSPO4* expression significantly decreased tumor growth and average tumor weight of LoVo xenografts in nude mice, compared with vector control (Fig. 2G). Taken together, these results suggested that RSPO4 acted as a tumor suppressor in tumor growth.

### RSPO4 mitigates tumor cell migration, invasion and stemness

To evaluate the effects of RSPO4 on tumor cell metastasis, we performed migration and invasion assays. Scratch wound healing assays showed that *RSPO4*-transfected cells had less efficient healing ability than vector control cells (Fig. S2E), suggesting a suppressive role of *RSPO4* on tumor cell migration. Transwell migration and Matrigel invasion assay also showed that *RSPO4*-transfected cells had significantly reduced ability of migration and invasion than vector controls (Fig. 2H).

EMT plays important roles in cancer cell invasion and metastasis 35. To explore whether EMT underlies RSPO4-mediated suppression of tumor cell migration and invasion, cell morphology and EMT markers were examined in tumor cells transfected with empty vector and *RSPO4* plasmid. We observed dramatic morphological alterations in *RSPO4*-transfected tumor cells, in which spindle-like and fibroblastic phenotype of mesenchymal cells were transmitted to cobblestone-like shape of epithelial cells (Fig. 2I). By immunofluorescence, increased epithelial marker E-cadherin and reduced mesenchymal marker vimentin were observed in *RSPO4*-transfected cells, compared with the vector control (Fig. 2J), indicating a reversed EMT phenotype. Consistent with this, Western blot detected increased level of E-cadherin, reduced levels of vimentin and fibronectin in tumor cells transiently and stably expressing *RSPO4* compared with vector control (Fig. 2K). Western blot also detected an opposite effect with *RSPO4* knockdown by siRNAs, including increased levels of N-cadherin, vimentin and fibronectin in tumor cells (Fig. 2L). Therefore, *RSPO4* expression can reverse the EMT program.

The activation of EMT results in quasi-mesenchymal cells which extensively reorganize the actin stress fibers 36. We hypothesized that RSPO4 may exert an effect on actin remodeling in cancer. Indeed, disruption of actin stress fibers increased levels of cortical F-actin, and reduced cell size was detected in *RSPO4*-transfected tumor cells, compared with vector control (Fig. 2M).

CSCs play an important role in cancer cell metastasis 61. Having demonstrated that loss of RSPO4 can push cells toward a more mesenchymal phenotype, we next performed sphere-formation assay to evaluate the effect of *RSPO4* expression on tumor cell stemness, as EMT is known to correlate with stem-like properties in both normal and cancer cell lines. We found that the sphere number and size of tumor cells was significantly reduced in *RSPO4*-expressing cell compared with vector control (Fig. 2N). Therefore, RSPO4 has a suppressive role on tumor cell migration, invasion and stemness.

### RSPO4 antagonizes Wnt/β-catenin signaling

**Figure 3:**
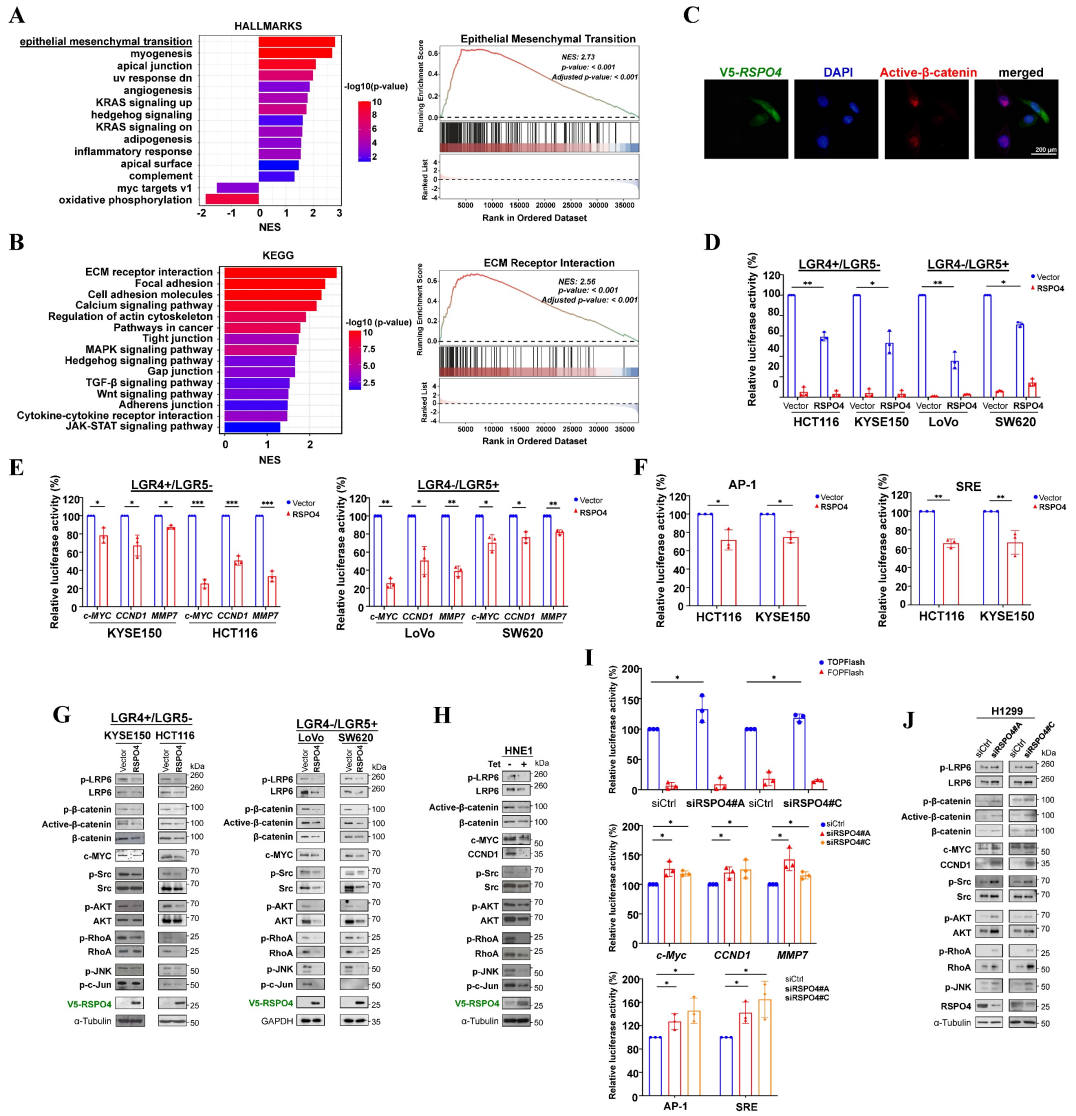
** RSPO4 antagonizes Wnt/β-catenin signaling in cancer cells.** (A) Gene set enrichment analysis of pathways in datasets of TCGA CRC patients (*Left*) and enrichment score of epithelial mesenchymal transition (*Right*). (B) Kyoto Encyclopedia of Genes and Genomes (KEGG) datasets of TCGA CRC patients (*Left*) and enrichment score of ECM receptor interaction (*Right*). (C) Immunofluorescent staining of nuclear β-catenin in cancer cells after 48 hrs transfection of *RSPO4* plasmid. Red, active β-catenin (unphosphorylated at Ser33/Ser37/Thr41); Green, RSPO4 protein stained by V5 antibody; Scale bar, 200 μm. (D) TOPflash/FOPflash luciferase reporter assay evaluated β-catenin/TCF activities in vector- and *RSPO4*-transfected cancer cells. (E) Transcriptional activities of *CCND1*, *c-MYC* and *MMP7* promoter were determined by luciferase reporter assay in vector- and *RSPO4*-transfected tumor cells (of either LGR4+/LGR5- or LGR4-/LGR5+ phenotype). (F) Transcriptional activities of AP-1 and SRE responsive element reporters were determined by luciferase reporter assay in vector- and *RSPO4*-transfected HCT116 and KYSE150 cells. (G) Western blot detection of the signaling alterations in canonical and non-canonical Wnt signaling in tumor cells transfected with *RSPO4* and vector. (H) Western blot detection of the signaling alterations in canonical and non-canonical Wnt signaling in HNE1 cells stably expressing *RSPO4* and vector. (I) Luciferase reporter assay detected β-catenin/TCF activities and transcriptional activities of Wnt target genes as well as AP-1 and SRE activities in H1299 cells with *RSPO4* knockdown by siRNAs. (J) Western blot examined the levels of the components of canonical and non-canonical Wnt signaling in H1299 cells with *RSPO4* knockdown by siRNAs. For D, E, F and I, n = 3 biologically independent replicates were examined over three independent experiments with similar results. Data are presented as mean values ± SD. Student's test was performed to obtain the P values.

To elucidate the molecular mechanisms underlying the tumor suppressive effects of *RSPO4*, we performed bioinformatic analysis of TCGA CRC dataset. Gene set enrichment analysis (GSEA) identified multiple enriched pathways, including EMT (Fig. 3A). Kyoto Encyclopedia of Genes and Genomes (KEGG) analysis likewise identified many enriched signaling pathways, including ECM receptor interaction, MAPK and Wnt signaling (Fig. 3B). Considering R-spondins are important regulators of Wnt signaling 62, 63, it would be possible that RSPO4-induced tumor suppressive effects were mediated by Wnt signaling. To test this hypothesis, we performed immunofluorescence staining and found that nuclear β-catenin levels were reduced in *RSPO4*-expressing tumor cells compared with vector control (Fig. 3C). By TOPflash reporter assay, we found that TOPflash activities were significantly reduced in *RSPO4*-expresing cells than that in vector controls (Fig. 3D), indicating that RSPO4 can induce the suppression on transcriptional activity of Wnt/β-catenin signaling. RSPO4 also induced significant suppression of the transcriptional activities of critical Wnt/β-catenin target genes *CCND1*, *c-MYC* and *MMP7*, which play important roles in tumor cell metastasis (Fig. 3E). As R-spondins can also regulate non-canonical Wnt signaling, we further checked the alteration of JNK/MAPK signaling, which is a key component of non-canonical Wnt signaling 7. We found that *RSPO4* expression significantly downregulated the promoter activity of AP-1 and SRE responsive element reporters (Fig. 3F), two major downstream targets of JNK 64, 65. To further confirm these effects, we performed Western blot and found that ectopic *RSPO4* expression led to decreased levels of LRP6 and phosphorylated LRP6 (Ser1490) (Fig. 3G and Fig. S3A). Levels of total-, phosphorylated- (Ser552) and active β-catenin (i.e. unphosphorylated at Ser33/Ser37/Thr41) as well as the downstream target gene (*c-MYC*) were also reduced in *RSPO4*-expressing tumor cells compared with vector control cells (Fig. 3G and Fig. S3A). Similar effects were observed in HNE1 cells stably expressing *RSPO4* (Fig. 3H). Treatment with RSPO4 CM or stimulation with exogenous recombinant human RSPO4 protein induced an inhibitory effect on Wnt/β-catenin signaling in cancer cells (Fig. S3B and S3C). Therefore, *RSPO4* expression results in the downregulation of both canonical and non-canonical Wnt signaling in cancer cells.

RhoA plays important roles in the coordinated assembly of stress fibers 66. At the molecular level, Western blot showed that *RSPO4* expression led to reduced phosphorylation of RhoA (Ser188) in tumor cells (Fig. 3G). We also examined the effects of *RSPO4* on upstream and downstream of RhoA signaling in stress fiber formation 67. Western blot showed that the phosphorylation of Src, AKT, ERK1/2, JNK and c-Jun was strongly suppressed by both transient (Fig. 3G) and stable *RSPO4* expression (Fig. 3H). Knockdown of *RSPO4* expression by siRNAs generated an opposite effect in tumor cells by luciferase reporter assay (Fig. 3I), leading to the activation of canonical and non-canonical Wnt signaling. Western blot confirmed that knockdown of *RSPO4* by siRNAs could promote both canonical and non-canonical Wnt signaling (Fig. 3J). Taken together, these results indicated that RSPO4 inhibited tumor cell proliferation and metastasis through antagonizing both canonical and non-canonical Wnt signaling.

### LGR4/5 and ZNRF3 are required for RSPO4-induced suppression of Wnt signaling

R-spondins have often been considered important potentiators of Wnt signaling. We wondered why RSPO4 antagonizes, rather than potentiates, Wnt signaling in tumor cells. Previous studies indicated that RSPO1 and RSPO2 suppress Wnt/β-catenin signaling in an LGR5, but not LGR4, -dependent manner 16, 33. R-spondin proteins bind to and co-internalize with LGR5 and LGR4, and LGR4 can play compensatory roles for LGR5 in Wnt signaling 33. Intriguingly, we found that RSPO4 suppressed Wnt/β-catenin signaling in both *LGR4* and *LGR5* expressing tumor cells (Fig. 3E and 3G, Fig. S4A). Therefore, we speculated that RSPO4 suppressed Wnt/β-catenin signaling possibly through interacting with either LGR4 or LGR5, which then stabilizes ZNRF3. ZNRF3 acts as a transmembrane E3 ubiquitin ligase that specifically mediates the multi-ubiquitination and degradation of Wnt receptors such as LRP6, as we observed that LRP6 protein expression was dramatically reduced in *RSPO4*-expressing cells compared with vector control (Fig. 3G and 3H).

**Figure 4:**
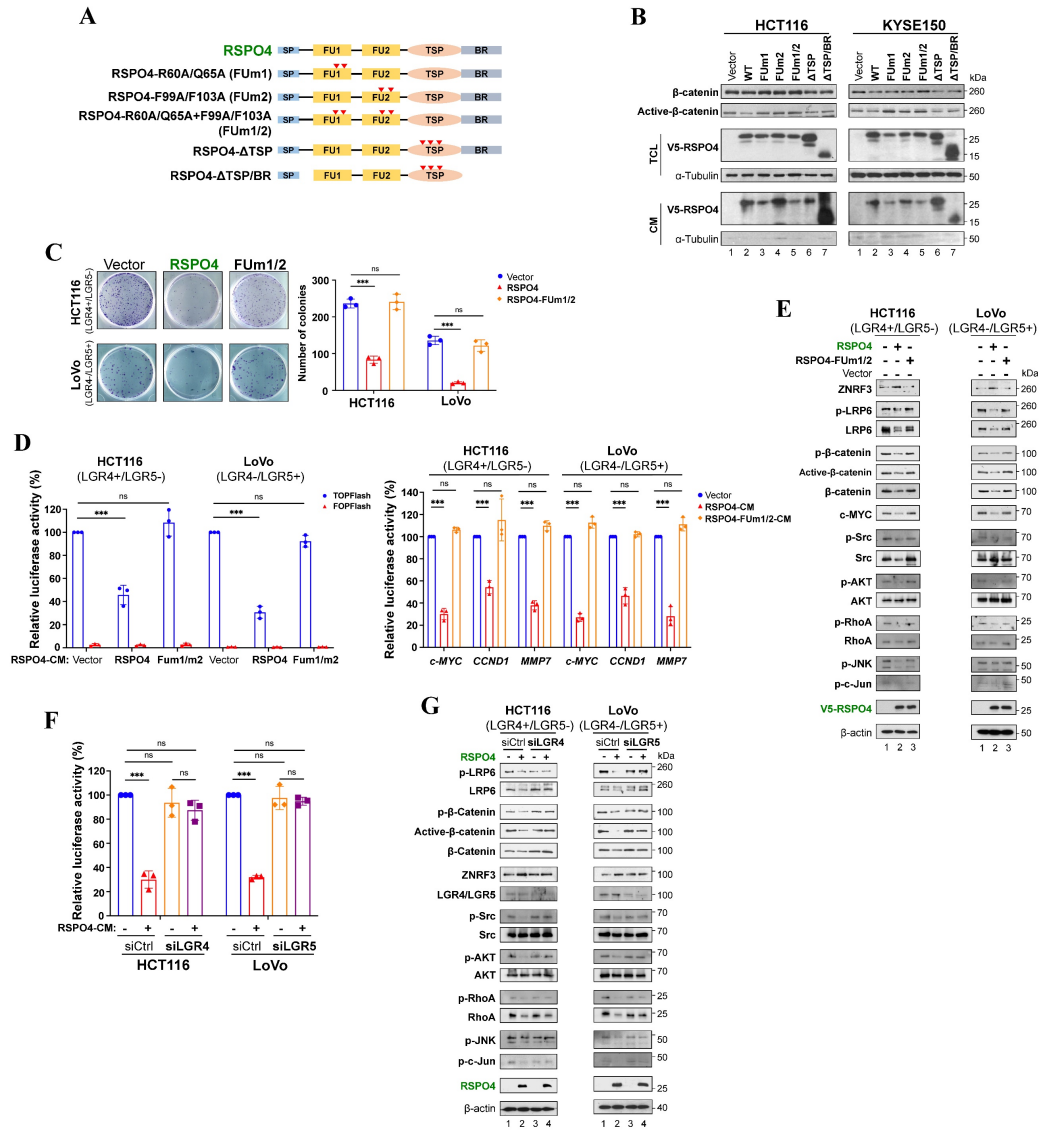
** RSPO4 suppresses of Wnt/β-catenin signaling in an LGR4/5 dependent manner.** (A) Schematic structure of RSPO4 protein and its mutants. (B) Western blot detected the β-catenin and active β-catenin level in HCT116 and KYSE150 cells transfected with vector-, *RSPO4*-WT, FUm1, FUm2, FUm1/2, ΔTSP and ΔTSP/BR. After 48 hrs transfection, cells were harvested for Western blot. (C) Colony formation assay in cancer cells transfected with vector, *RSPO4* and FUm1/2. (D) TOPflash/FOPflash luciferase reporter assay in vector-, *RSPO4*- and FUm1/2-transfected tumor cells (*left*). Transcriptional activities of *CCND1*, *c-MYC* and *MMP7* promoter reporter in vector-, *RSPO4*- and FUm1/2-transfected cancer cells (*right*). (E) Western blot detected the signaling alterations in canonical and non-canonical Wnt signaling in cancer cells transfected with vector, *RSPO4* and FUm1/2. (F) TOPflash/FOPflash luciferase reporter assay detected the transcriptional activity of β-catenin in cancer cells with knockdown of *LGR4* or *LGR5* by siRNAs. (G) Western blot detected the signaling alterations in canonical and non-canonical Wnt signaling in cancer cells with vector and *LGR4* or *LGR5* knockdown by siRNAs. For C, D and F, n = 3 biologically independent replicates were examined over three independent experiments with similar results. Data are presented as mean values ± SD. Student's test was performed to obtain the P values.

R-spondins bind to LGR4/5 via the FU2 domain, and interact with ZNRF3/RNF43 through its FU1 domain 68, 69. To confirm that LGR4/5 and ZNRF3/RNF43 are required for RSPO4-induced suppression of Wnt/β-catenin signaling, we constructed mutant variants at the most conserved residues of RSPO4 protein which inactivate the function of FU1, FU2, TSP and BR domains, respectively (Fig. 4A) 68-70. Western blot detected that RSPO4 with inactivation of FU1 (FUm1) and FU2 (FUm2) domain alone or combination (FUm1/2) (*column 3-5*, Fig. 4B), rather than TSP and BR domain (*column 6* and *7*, Fig. 4B), lost the ability to decrease the levels of β-catenin and active β-catenin, indicating that these mutants lost their inhibitory effects on Wnt/β-catenin signaling. Moreover, inactivation of FU1 and FU2 domain (FUm1/2) lost the suppressive effects on tumor cell clonogenicity (Fig. 4C). These data suggested that RSPO4-induced Wnt/β-catenin signaling depends on FU1 and FU2 domains, but not the TSP and BR domains. Consistently, luciferase reporter assay showed that RSPO4 FUm1/2 mutant lost the inhibitory effect on Wnt/β-catenin signaling and target genes in both LGR4+/LGR5- and LGR4-/LGR5+ tumor cell lines (Fig. 4D). Western blot showed that cells with *RSPO4* FUm1/2 expression presented reduced level of ZNRF3, and increased levels of LRP6, β-catenin and c-MYC compared with wildtype *RSPO4* (Fig. 4E). To further confirm this, we depleted the expression of *LGR4* and *LGR5* by siRNAs, and found that after the knockdown, RSPO4 lost its inhibitory effect on Wnt/β-catenin signaling in both LGR4+/LGR5- and LGR4-/LGR5+ tumor cell lines (Fig. 4F and 4G), and ZNRF3 protein was unable to accumulate in the presence of RSPO4 (*lane 2* and *lane 3*, Fig. 4G). Thus, either LGR4 or LGR5 is required for RSPO4-induced accumulation or stabilization of ZNRF3. Thus, LGR4/5 and ZNRF3 are required for RSPO4-induced suppression of Wnt/β-catenin signaling in cancer cells.

We also assessed whether *LGR4/5* and *ZNRF3* expression were regulated at the transcriptional level (Fig. S4A), so there might be a regulatory feedback effect in RSPO4-induced suppression. We treated tumor cells with recombinant human RSPO4 protein and observed its effect at different time points and found that RSPO4 induced an initial increase of β-catenin level within the first hour followed by an attenuated response thereafter (Fig. S4B). We screened mRNA expression of *LGR4* and *ZNRF3* at the corresponding time points. Semi-RT-PCR results indicated that mRNA expression of *LGR4* and *ZNRF3* is reversely corresponding with protein level of β-catenin and mRNA level of *c-MYC* with the final upregulation of *LGR4* and *ZNRF3* (Fig. S4C). Thus, RSPO4 induces the initial downregulation and final upregulation of *LGR4/5* and *ZNRF3* expression, forming a negative feedback loop.

### RSPO4 recruits LGR4/5 to prevent ubiquitin-proteasome mediated degradation of ZNRF3

**Figure 5:**
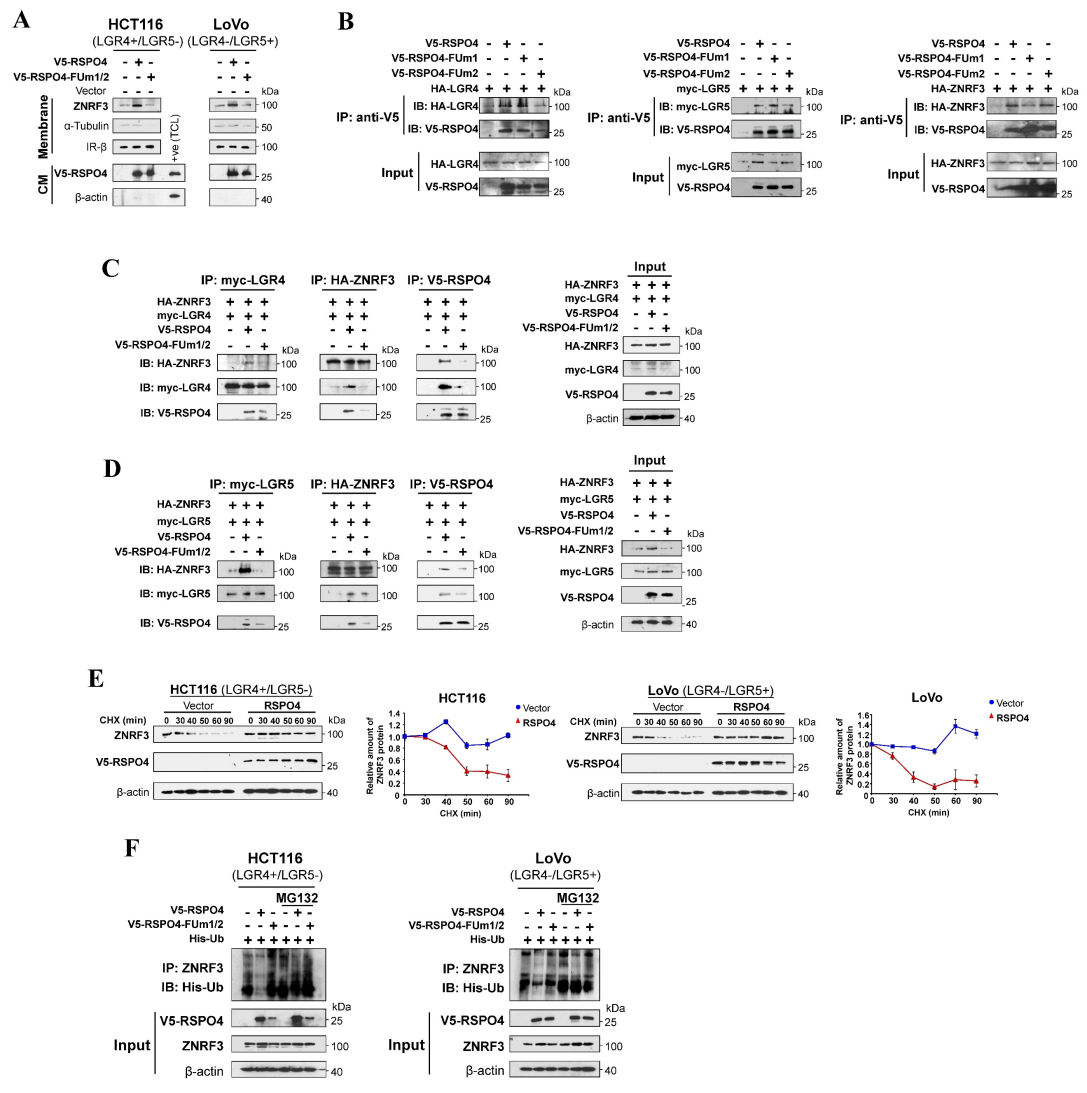
** RSPO4 recruits LGR4/5 to prevent the ubiquitin-proteasome mediated degradation of ZNRF3.** (A) Western blot detection of membrane ZNRF3 in LGR4+/LGR5- and LGR4-/LGR5+ cancer cells transfected with vector, *RSPO4* and FUm1/2. Membrane ZNRF3 was isolated after 48 hrs transfection of vector, *RSPO4* and FUm1/2. (B) V5-epitope-tagged *RSPO4*, FUm1 and FUm2 were co-transfected with HA-epitope-tagged LGR4 in HEK293 cells (*left panel*). V5-epitope-tagged *RSPO4*, FUm1 and FUm2 were co-transfected with myc-epitope-tagged *LGR5* in HEK293 cells (*middle panel*). V5-epitope-tagged *RSPO4*, FUm1 and FUm2 were co-transfected with HA-epitope-tagged ZNRF3 in HEK293 cells (*right panel*). After 48 h transfection, membrane proteins were prepared and immunoprecipitated by using V5 antibody. Immunoblotting was probed by V5, myc and HA antibody. (C) V5-epitope-tagged *RSPO4* and FUm1/2 were co-transfected with HA-epitope-tagged ZNRF3 and myc-epitope-tagged LGR4 in HEK293 cells. After 48 h transfection, membrane proteins were prepared and immunoprecipitated by using myc (*left panel*), HA (*middle panel*) and V5 (*right panel*) antibody, respectively. Immunoblotting was probed by V5, myc and HA antibody. The input control was shown at the most right. (D) V5-epitope-tagged *RSPO4* and FUm1/2 were co-transfected with HA-epitope-tagged *ZNRF3* and myc-epitope-tagged *LGR5* in HEK293 cells. After 48 h transfection, membrane proteins were prepared and immunoprecipitated by using myc (*left panel*), HA (*middle panel*) and V5 (*right panel*) antibody, respectively. Immunoblotting was probed by V5, myc and HA antibody. The input control was shown at the most right. (E) Cycloheximide (CHX)-chase assay for the half-life of ZNRF3 in HCT116 and LoVo cells. HCT116 (*left panel*) and LoVo (*right panel*) cells with *RSPO4* or vector expression are treated with CHX (20 μg/ml) for the indicated time points, and Western blot with indicated antibodies. (F) RSPO4 decreases ZNRF3 ubiquitination. *RSPO4* and FUm1/2 were co-transfected with His-Ub plasmid into HCT116 and LoVo cells followed by treatment of 10 μM MG132 for 6 h. Membrane proteins were prepared and immunoprecipitated by using ZNRF3 antibody. Immunoblotting was probed by His antibody.

We wonder how RSPO4 regulates the protein level of ZNRF3. As an E3 ubiquitin ligase, ZNRF3 is the final effector of the RSPO4-LGR4/5-ZNRF3 axis when targeting Wnt receptors 60. In cells with RSPO2-induced suppression of Wnt/β-catenin signaling, LGR5 is required for the accumulation or stabilization of membrane ZNRF3 16. To find out the effect of *RSPO4* expression on ZNRF3, we isolated the membrane protein after transfecting *RSPO4* and its FUm1/2 mutant into HEK293T cells. Western blot showed that membrane ZNRF3 accumulated remarkably in *RSPO4*-expressed cells compared with vector control in LGR4+/LGR5- or LGR4-/LGR5+ cell lines (Fig. 5A), suggesting that RSPO4 stabilizes membrane ZNRF3. Expression of FUm1/2 mutant reduced ZNRF3 to the level as vector control (Fig. 5A), suggesting that RSPO4 might be required for ZNRF3 accumulation.

To confirm RSPO4 directly interacts with LGR4/5 and ZNRF3, we performed co-immunoprecipitation using V5 antibody and found that FUm2 lost the ability to interact with LGR4 (Fig. 5B, *left panel*) and LGR5 (Fig. 5B, *middle panel*), and FUm1 lost the ability to interact with ZNRF3 (Fig. 5B, *right panel*). By pull down with myc (LGR4), HA (ZNRF3) and V5 (RSPO4) antibodies individually, co-immunoprecipitation confirmed that RSPO4 directly interacts with LGR4 and ZNRF3, and FUm1/2 mutation lost this interacting ability (Fig. 5C). By using myc (LGR5), HA (ZNRF3) and V5 (RSPO4) antibodies, co-immunoprecipitation confirmed that RSPO4 directly interacts with LGR5 and ZNRF3, and FUm1/2 mutation lost this interacting ability (Fig. 5D). Therefore, these data suggested that RSPO4 directly interacts with LGR4/5 and ZNRF3, and mutations of FU1 and FU2 domain lost the ability to interact with ZNRF3 and LGR4/5, respectively.

To further investigate the effect of *RSPO4* expression on ZNRF3 at the protein level, we performed ZNRF3 degradation assay with protein synthesis inhibitor cycloheximide (CHX) in HEK293T cells transfected with empty vector or *RSPO4*. The half-life of endogenous ZNRF3 protein was dramatically extended in *RSPO4*-expressing cells compared with vector control in both LGR4+/LGR5- and LGR4-/LGR5+ cancer cells after CHX treatment (Fig. 5E), indicating that *RSPO4* expression prevents the degradation of ZNRF3, which resulted in the accumulation or stabilization of ZNRF3. Many proteins undergo modification of ubiquitylation followed by proteasome-mediated degradation 71. We examined whether ubiquitylation contributed to the degradation of ZNRF3. We treated *RSPO4*- and FUm1/2- expressing tumor cells with MG132, a typical proteasome inhibitor. We found that *RSPO4* expression dramatically inhibit ZNRF3 ubiquitylation (Fig. 5F), while FUm1/2 mutant lost this inhibitory effect, implying LGR4 or LGR5 is required for the inhibition of ZNRF3 ubiquitylation. MG132 treatment dramatically increased the ubiquitylation level of ZNRF3 after RSPO4 expression (Fig. 5F), suggesting that ZNRF3 underwent ubiquitin-proteasome mediated degradation and RSPO4 prevented the occurrence of this process. Taken together, these data suggested that RSPO4 recruits LGR4/5 to stabilize ZNRF3 through preventing its ubiquitin-proteasome mediated degradation.

### RSPO4 suppresses tumor cell migration, invasion and stemness through inhibiting Wnt signaling

**Figure 6:**
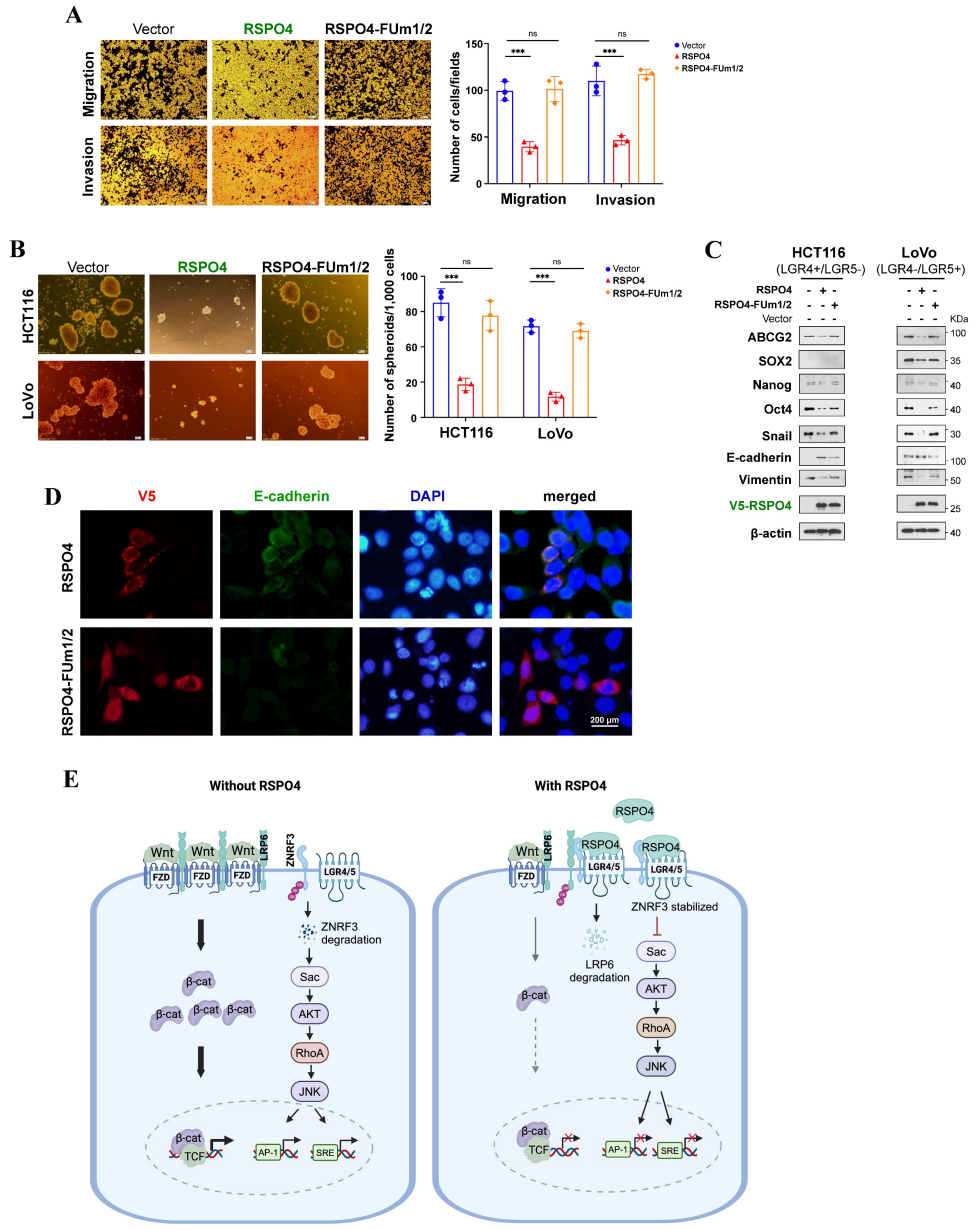
** RSPO4 mitigate cancer cell migration, invasion, stemness through Wnt/β-catenin signaling.** (A) Transwell migration and invasion assay of HCT116 cells transfected with empty vector, *RSPO4* and FUm1/2 plasmid. (B) Sphere-forming assays evaluated the stemness of cancer cells transfected with empty vector, *RSPO4* and FUm1/2 plasmid. Scale bar, 100μm. (C) Western blot detected the expression levels of EMT and stem cell markers in HCT116 and LoVo cells transfected with empty vector, *RSPO4* and FUm1/2 plasmid. (D) Indirect immunofluorescence staining of E-caherin in empty vector-, *RSPO4*- and FUm1/2 transfected KYSE150 cells. Original magnification, ×400. Scale bar, 200μm. (E) Schematic diagram illustrates the role of RSPO4, functioning as a tumor suppressor through antagonizing Wnt/β-catenin signaling dependent on LGR4/5 and ZNRF3 by forming a negative feedback loop. Diagram was created with BioRender. For A and B, n = 3 biologically independent replicates were examined over three independent experiments with similar results. Data are presented as mean values ± SD. Student's test was performed to obtain the P values.

We assessed whether RSPO4 mitigates tumor cell migration, invasion, stemness through inhibiting Wnt signaling. We found that *RSPO4* FUm1/2 mutant lost its inhibitory effect on migration and invasion of both LGR4+/LGR5- and LGR4-/LGR5+ tumor cells (Fig. 6A). By sphere formation assay, we found that FUm1/2 mutant expression lost the inhibitory effect of *RSPO4* on cancer cell stemness of both LGR4+/LGR5- and LGR4-/LGR5+ tumor cells (Fig. 6B). Moreover, FUm1/2 mutant expression also lost the inhibitory effect of *RSPO4* on stem cell marker expression, and Western blot indicated that FUm1/2 mutant was unable to downregulate the expression of EMT markers (Fig. 6C). Consistent with this, immunofluorescence showed that FUm1/2 mutant expression lost the ability to induce E-cadherin expression (Fig. 6D). Therefore, LGR4/5 and ZNRF3 are required for the suppressive effect of *RSPO4* expression on tumor cell migration, invasion and stemness, and RSPO4 exerts these effects through suppressing Wnt signaling.

## Discussion

Multiple mechanisms have been reported to regulate the expression of RSPO members in human cancers. Of these, promoter CpG methylation was found to inactivate *RSPO1*, *RSPO2* and *RSPO3* in acute lymphocytic leukemia, CRC, and cholangiocarcinoma, respectively 16, 28, 29. In this study, through methylomic study and database mining, we identified another member of the R-spondin family, *RSPO4*, as a TSG candidate inactivated by promoter CpG methylation in multiple carcinomas in a tumor-specific way. Thus, promoter CpG methylation tends to be a common mechanism inactivating RSPO member expression in cancers, making them potential cancer biomarkers and therapeutic targets.

R-spondins were initially discovered as Wnt agonists which promotes cancer development and metastasis 10. Recently, RSPO2 was identified as a Wnt antagonist, but not agonist, through inhibiting Wnt/β-catenin signaling in CRC 16. In this study, we found that RSPO4 inhibited tumor cell proliferation, metastasis and stemness through suppressing both canonical and non-canonical Wnt/β-catenin signaling, indicating RSPO4 as a Wnt antagonist. In CRC, RSPO2 antagonizes Wnt/β-catenin signaling dependent on LGR5, instead of LGR4. We found that RSPO4 inhibited Wnt/β-catenin signaling dependent on either LGR4 or LGR5, indicating the functional complexity of RSPO members in human carcinogenesis.

Previous studies showed that RSPO1 and RSPO2 could potentiate Wnt/β-catenin signaling in the absence of LGR5 expression 16, 33. Such effect was not observed in our study, possibly due to the residual expression of endogenous LGR5 even after the siRNA knockdown (Fig. 4G). We propose a RSPO4-induced Wnt/β-catenin signaling feedback model (Fig. 6E), the net output of which is the attenuation of Wnt/β-catenin signaling in the presence of RSPO4. In the absence of RSPO4, ZNRF3 is highly ubiquitinated and degraded and so remains at low protein level, and Wnt/β-catenin signaling is normally active. When present, RSPO4 recruits LGR4/5 and stabilizes ZNRF3, which acts as an E3 ubiquitin ligase interacting and internalizing Wnt receptors such as LRP6, and leading to their degradation, then Wnt/β-catenin signaling is finally attenuated. Therefore, RSPO4 shares similarities with RSPO2 in the mechanism of tumor suppression but with obvious difference 16.

Our model is different from the regulatory model of R-spondins Hao HX *et al.* initially proposed 60, in which ZNRF3 alone ubiquitinates and degrades Wnt receptors in the absence of R-spondins. In the presence, R-spondins form complexes with LGR4/5 and ZNRF3, which then undergoes internalization and degradation. ZNRF3 is thus cleared from the membrane. Without ZNRF3 and its ubiquitination, Wnt receptors stay at high level, which therefore potentiates downstream Wnt/β-catenin signaling. However, in our model, ZNRF3 alone was not able to ubiquitinate and degrade Wnt receptors, but underwent degradation itself. LGR4 or LGR5 is required when ZNRF3 acts as an E3 ubiquitin ligase targeting Wnt receptors. The opposite effects of R-spondins in two models might be explained by the varying cellular context and requires further exploration. In our model, RSPO4 induces a transient activation and final inhibition of Wnt/β-catenin signaling. Conversely, RSPO4 induces an initially lower and final higher mRNA expression of *LGR4* and *ZNRF3* than the baseline expression level. Therefore, RSPO4 antagonizes Wnt/β-catenin signaling by forming a negative feedback loop.

Cancer metastasis involves the delamination of cells from primary tumors, possibly through EMT program. The EMT process involves protein dynamics resulting in complex alterations in cell behaviors, such as reduced cell-cell adhesion, enhanced motility and remodeling of actin cytoskeleton 72. Indeed, we found that RSPO4 regulated the assembly of actin cytoskeleton through non-canonical and canonical Wnt signaling. Wnt/β-catenin signaling is one of the most important activators of EMT program 35. In this study, RSPO4 reversed EMT through suppressing Wnt/β-catenin signaling which either regulated EMT-transcription factors (e.g. SNAI1) or other Wnt/β-catenin-targeted genes such as *MMP7* 36. However, mechanistic differences of R-spondin members might exist in regulating EMT program. For example, RSPO2 suppressed EMT in CRC by counteracting Wnt5a/Fzd7-driven non-canonical Wnt signaling 17.

Cancer stem cells (CSCs) usually refer to tumor cells with self-renewal capacity and multi-differentiation potential 61. The EMT program enables the generation of CSCs at different steps of the metastatic process including metastatic colonization. With their ability to initiate tumors and cellular plasticity, CSCs are able to repopulate metastatic outgrowths 73. Therefore, CSCs appear to be major sources of therapeutic resistance and tumor relapse. Wnt/β-catenin signaling is one of many important pathways regulating cancer stemness and malignant progression through regulating EMT transcription factors 74. R-spondin/LGR5/ZNRF3 axis enhance Wnt/β-catenin activity and thereby likely promote stem cell properties 75. Indeed, LGR5 has been reported to be a CSC marker in many malignancies 76, 77. Stemness genes (e.g. *SOX2*,* NANOG*,* OCT4*,* SNAI1*,* ABCG2 etc*.) are important promoters of stemness and metastasis in different cancer types 78. In this study, we found that RSPO4 suppressed EMT and tumor cell stemness through Wnt/β-catenin signaling in an LGR4/5 dependent manner. Moreover, RSPO4 can downregulate the expression of many EMT transcription factors such as *SNAI1* and stem cell markers such as *ABCG2*, *SOX2*, *NANOG* and *OCT4*. Therefore, RSPO4 provides another therapeutic target for inhibiting EMT and cancer stemness.

RSPO4 functions as a tumor suppressor by antagonizing canonical and non-canonical Wnt signaling in an LGR4/5- and ZNRF3- dependent manner. Our study emphasizes the functional complexity of RSPO family members in the context of human cancers. The tumor-specific promoter CpG methylation of* RSPO4* makes it a potential cancer biomarker and therapeutic target.

## Supplementary Material

Supplementary figures and tables.

## Figures and Tables

**Table 1:** The association between *RSPO4* methylation and clinicopathological features of CRC patients (TCGA, Firehose Legacy)

Clinical characteristic	*RSPO4* methylation		*P*-value
	No (n=94)	Yes (n=298)	
Gender			
Female	50	130	0.132
Male	44	168	
Diagnosis Age (years)			
<=55	34	65	**0.008**
>55	60	233	
Weight (kg)			0.708
<= 60	12	36	
>= 60 & <= 100	52	168	
> 100	13	31	
Histological Type			**0.001**
Colon Adenocarcinoma	60	192	
Colon Mucinous Adenocarcinoma	1	38	
Rectal Adenocarcinoma	31	59	
Rectal Mucinous Adenocarcinoma	0	6	
TNM stage			0.351
Stage I	13	42	
Stage II	30	113	
Stage III	30	90	
Stage IV	18	36	
Lymph node stage			0.164
N0	47	166	
N1	32	71	
N2	15	57	
Infiltration depth			0.875
T1	2	9	
T2	15	40	
T3	66	206	
T4	11	40	
Metastasis stage			0.458
M0	60	206	
M1	16	37	
Mx	17	48	
Tumor size (cm)			**0.035**
>= 2.0	2	11	
>0.5 & <2.0	30	136	
<= 0.5	18	34	
*KRAS* mutation			0.261
No	10	19	
Yes	5	23	
*BRAF* mutation			0.982
No	6	26	
Yes	0	3	

**Table 2:** Summary of *RSPO4* methylation in epithelial cell lines and primary tumors

	Cell lines (% methylated)	Tumors (% methylated)
Carcinoma		
Nasopharyngeal	100% (5/5)	96% (22/23)
Esophageal	44% (8/18)	39% (24/62)
Lung	38% (3/8)	-
Colorectal	82% (9/11)	45% (5/11)
Bladder	67% (2/3)	-
Ovary	33% (1/3)	-
Brain	100% (2/2)	-
Immortalized normal epithelial cell lines		
NP69, NE1, NE3	0% (0/3)	
Normal epithelial cell lines		
Het-1A	0% (0/1)	

**Table 3:** Summary of *RSPO4* alterations in multiple types of human cancer from TCGA datasets

Cancer Type	Sample number^a^ (n^b^)	Mutation	Homozygous deletion	Heterozygous deletion	Methylation^c^	Cohort
Esophageal Carcinoma	183 (186)	1.6% (n=3)	-	14.2% (n=26)	70.5% (n=129)	TCGA, Firehose Legacy
Lung Squamous Cell Carcinoma	367 (511)	-	0.3% (n=1)	9.0% (n=33)	53.1% (n=195)	TCGA, Firehose Legacy
Colorectal Adenocarcinoma	392 (640)	0.3% (n=1)	0.5% (n=2)	20.2% (n=79)	76.0% (n=298)	TCGA, Firehose Legacy
Head and Neck Squamous Cell Carcinoma	279 (279)	0.4% (n=1)	-	12.2% (n=34)	81.7% (n=228)	TCGA, Nature 2015 79
Brain Lower Grade Glioma	510 (530)	0.2% (n=1)	0.2% (n=1)	1.9% (n=10)	14.7% (n=75)	TCGA, Firehose Legacy
Bladder Urothelial Carcinoma	129 (131)	-	-	8.5%(n=11)	17.8 % (n=23)	TCGA, Nature 2014 80

Data extracted from cBio (http://www.cbioportal.org/). ^a^ data removed with β-value = “NA”; ^b^ total sample size; ^c^ β-value>0.3 is considered as methylation, and methylation is not mutual exclusive with mutation and deletion.

## Abbreviations

RSPO4: R-spondin 4
TSGs: tumor suppressor genes
YAP: Yes-associated protein
CRC: colorectal cancer
LUAD: lung adenocarcinoma
FU: furin-like cysteine-rich domains
TSP: thrombospondin domain
BR: basic region
EMT: epithelial-mesenchymal transition
CSC: cancer stem cell
ATCC: American Type Culture Collection
MSP: methylation-specific PCR
BGS: bisulfite genomic sequencing
CM: conditioned media
SD: standard deviation
MeDIP: methylated DNA immunoprecipitation
dRRBS: double-enzyme reduced representation bisulfite sequencing
NPC: nasopharyngeal cancer
ESCC: esophageal squamous cell carcinoma
Lung Ca: lung cancer
CRC: colorectal cancer
Blad Ca: bladder cancer
OvCa: ovary cancer
READ: rectum adenocarcinoma
CFA: colony formation assay
PARP: poly (ADP-ribose) polymerase
GSEA: gene set enrichment analysis
KEGG: Kyoto Encyclopedia of Genes and Genomes
CHX: cycloheximide
