# Supplementary material for: RSPO4 exerts tumor suppression through antagonizing canonical and non-canonical Wnt signaling
Source: Int J Biol Sci. 2026 Jan 1;22(2):1016–35. doi: 10.7150/ijbs.124734 (PMC12781852; doi:10.7150/ijbs.124734)
Supplement: Supplementary file 1 — Supplementary figures and tables. [file ijbsv22p1016s1.pdf]

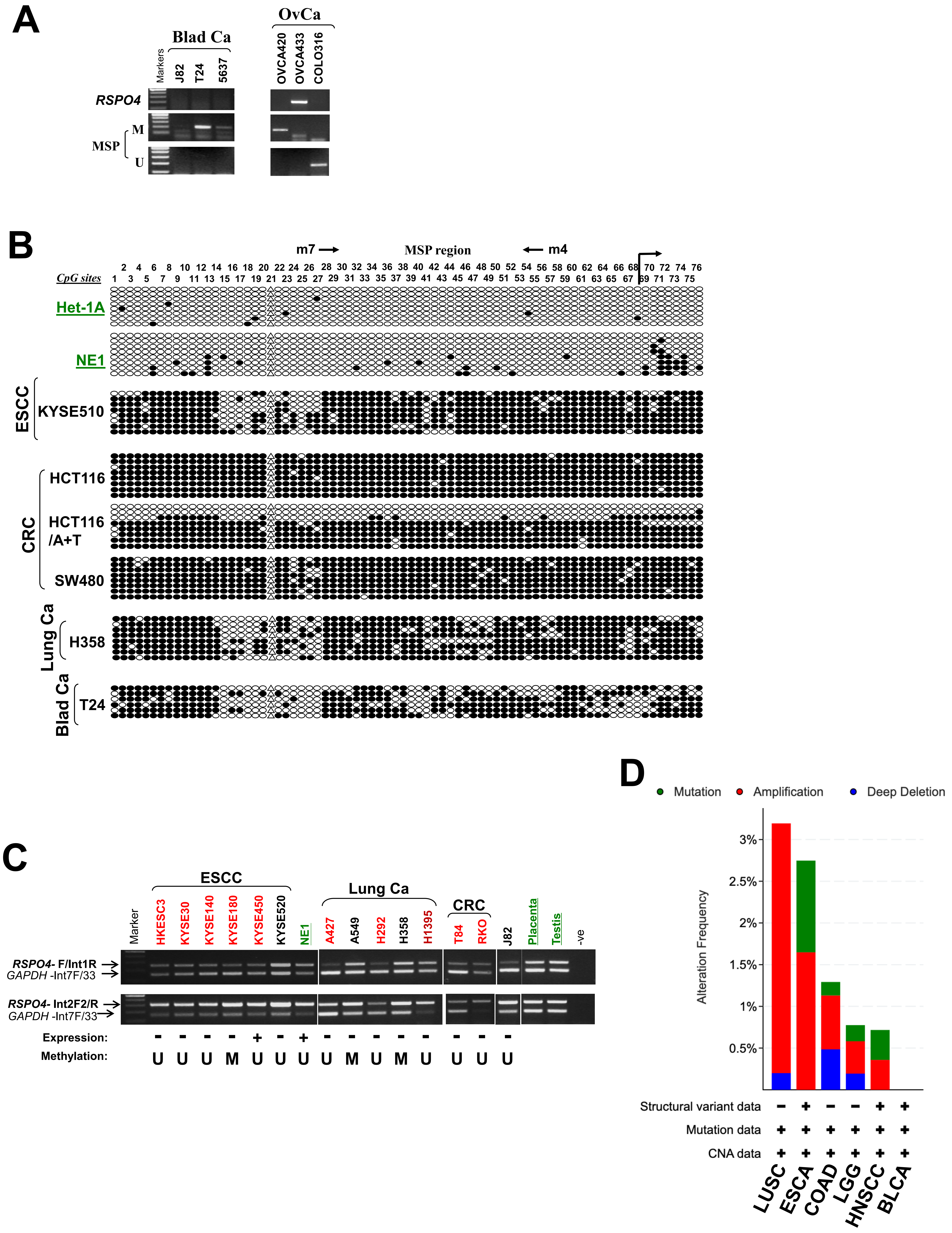
A
Blad Ca
OvCa
Markers
J82
T24
5637
OVCA420
OVCA433
COLO316
RSPO4
MSP
M
U
B
m7
MSP region
m4
CpG sites
Het-1A
NE1
ESCC
KYSE510
CRC
HCT116
HCT116 /A+T
SW480
Lung Ca
H358
Blad Ca
T24
C
ESCC
Lung Ca
CRC
Marker
HKESC3
KYSE30
KYSE140
KYSE180
KYSE450
KYSE520
NE1
A427
A549
H292
H358
H1395
T84
RKO
J82
Placenta
Testis
-ve
RSPO4- F/Int1R
GAPDH -Int7F/33
RSPO4- Int2F2/R
GAPDH -Int7F/33
Expression: - - - - + - + - - - - - - - -
Methylation: U U U M U U U U M U M U U U U
D
Mutation
Amplification
Deep Deletion
Alteration Frequency
3%
2.5%
2%
1.5%
1%
0.5%
Structural variant data - + - - + +
Mutation data + + + + + +
CNA data + + + + + +
LUSC
ESCA
COAD
LGG
HNSCC
BLCA

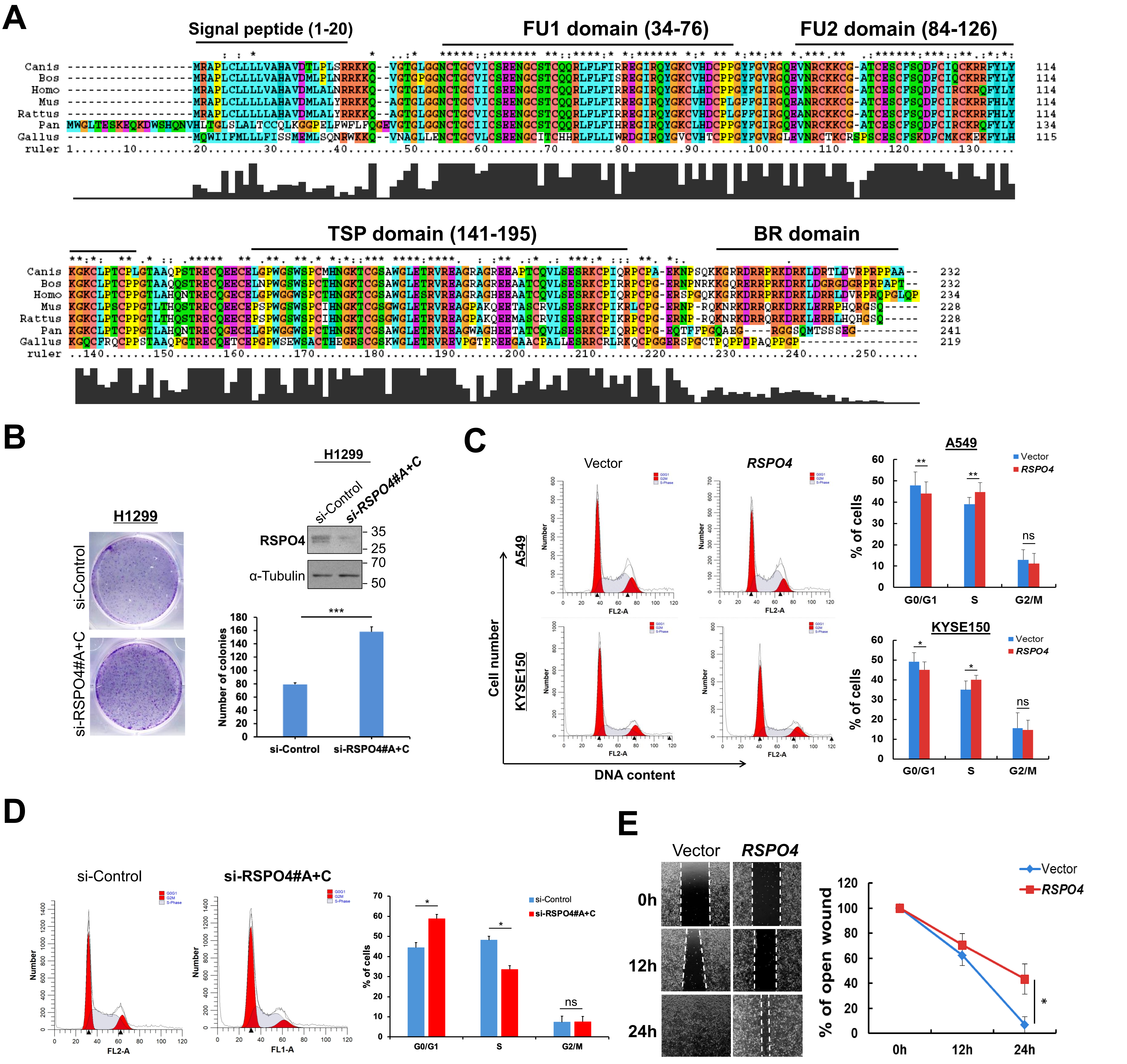
A
Signal peptide (1-20)
FU1 domain (34-76)
FU2 domain (84-126)
TSP domain (141-195)
BR domain
Canis
Bos
Homo
Mus
Rattus
Pan
Gallus
ruler
B
H1299
si-Control
si-RSPO4#A+C
RSPO4
α-Tubulin
35
25
70
50
Number of colonies
***
C
Vector
RSPO4
A549
KYSE150
Cell number
DNA content
FL2-A
Number
% of cells
G0/G1
S
G2/M
ns
D
si-Control
si-RSPO4#A+C
FL1-A
E
0h
12h
24h
% of open wound

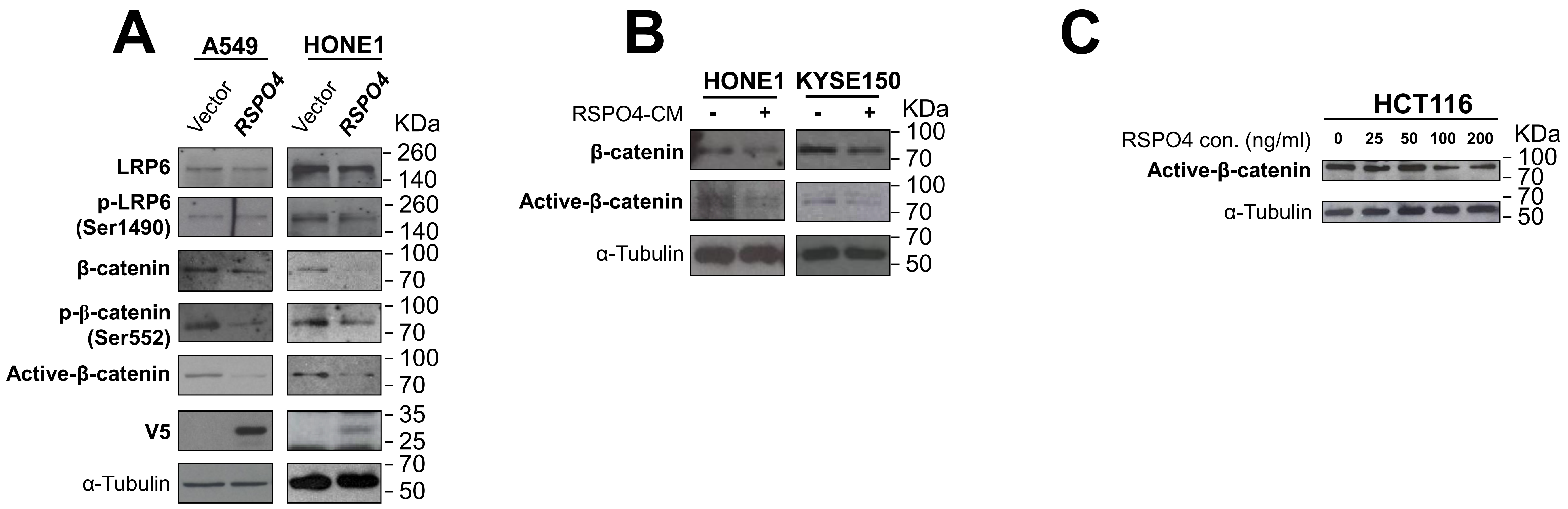
A
A549
HONE1
Vector
RSPO4
Vector
RSPO4
KDa
LRP6
p-LRP6 (Ser1490)
β-catenin
p-β-catenin (Ser552)
Active-β-catenin
V5
α-Tubulin
260
140
260
140
100
70
100
70
100
70
35
25
70
50
B
HONE1
KYSE150
RSPO4-CM
-
+
-
+
KDa
β-catenin
Active-β-catenin
α-Tubulin
100
70
100
70
70
50
C
HCT116
RSPO4 con. (ng/ml)
0
25
50
100
200
KDa
Active-β-catenin
α-Tubulin
100
70
70
50

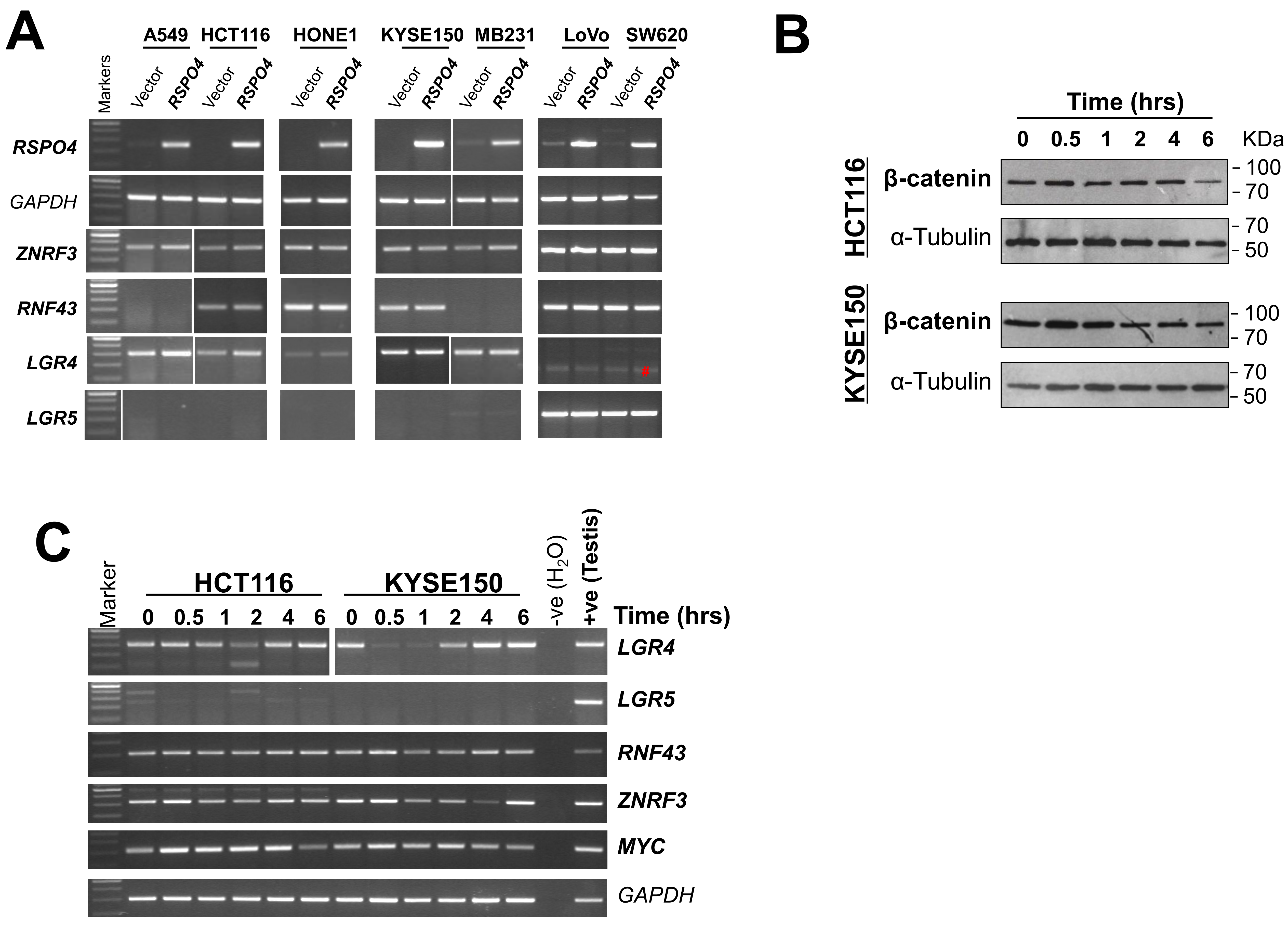
A
A549
HCT116
HONE1
KYSE150
MB231
LoVo
SW620
Markers
Vector
RSPO4
RSPO4
GAPDH
ZNRF3
RNF43
LGR4
LGR5
B
Time (hrs)
0
0.5
1
2
4
6
KDa
HCT116
β-catenin
α-Tubulin
KYSE150
β-catenin
α-Tubulin
100
70
50
C
Marker
HCT116
KYSE150
-ve (H2O)
+ve (Testis)
Time (hrs)
0
0.5
1
2
4
6
LGR4
LGR5
RNF43
ZNRF3
MYC
GAPDH

Supplementary Figure 1. Inactivation of *RSPO4* expression is mediated by promoter CpG methylation.
(A) RT-PCR and MSP detected the mRNA expression and promoter methylation of *RSPO4* in bladder and ovary cancer cell lines. M, methylated; U, unmethylated. Blad Ca, bladder cancer; OVCA, ovary cancer.
(B) BGS analysis of the *RSPO4* promoter in representative cancer cell lines.
(C) Representative deletion analysis of *RSPO4* in multiple carcinoma cell lines, normal tissue and epithelial cell lines (underlined). *RSPO4* deletion was examined by multiplex differential genomic DNA-PCR using primers targeting a region spanning exons 1 and 3 using *GAPDH* as an internal control. Ca, carcinoma; ESCC, esophageal squamous cell carcinoma; CRC, colorectal cancer. The expression and methylation status of *RSPO4* in each sample is also shown in bottom panels. +, expressed; -, downregulated/silenced; M, methylated; U, unmethylated.
(D) *RSPO4* is genetically altered in a variety of human cancers, including mutation, deletion and amplification. LUSC, Lung Squamous Cell Carcinoma; ESCA, Esophageal Carcinoma; COAD, Colorectal Adenocarcinoma; LGG, Brain Lower Grade Glioma; HNSCC, Head and Neck Squamous Cell Carcinoma; BLCA, Bladder Urothelial Carcinoma. The TCGA datasets in this figure are the same as in Table 3.

Supplementary Figure 2. *RSPO4* encodes a secreted protein which inhibits tumor cell clonogenicity.
(A) Amino acid sequence alignment of R-spondin family members.
(B) Colony formation assay in H1299 cells with knockdown of *RSPO4* by siRNAs. After 24hrs transfection, cells were seeded into 6 well plates. After incubation for 3~5 days, cells were fixed, stained and counted.
(C) Cell cycle analysis using PI staining by flow cytometry. Representative cell cycle histograms of A549 and KYSE150 cells with *RSPO4* expression show significant decrease in G0/G1 populations as well as significant increase in S phase population, compared to controls. Bar diagram compares variations in cell distribution percentage in each phase of cell cycle of A549 and KYSE150 cells.
(D) Cell cycle analysis using PI staining by flow cytometry. Representative cell cycle histograms of H1299 cells with knockdown of *RSPO4* by siRNAs show significant increase in G0/G1 populations as well as significant decrease in S phase population, compared to controls. Bar diagram compares variations in cell distribution percentage in each phase of cell cycle of H1299 cells.
(E) Wound healing assay in HONE1 cells with vector- or *RSPO4*-transfection.
For B, C, D and E, n = 3 biologically independent replicates were examined over three independent experiments with similar results. Data are presented as mean values ± SD. For B, C and D, Student's test was performed to obtain the P values. For E, statistical analysis is performed by one-way ANOVA.

Supplementary Figure 3. RSPO4 antagonizes Wnt/β-catenin signaling.
(A) Western blot detected the protein level of β-catenin in A549 and HONE1 cells transfected with vector and *RSPO4*.
(B) Western blot detected the protein level of β-catenin in HONE1 and KYSE150 cells with RSPO4 CM treatment for 24 hrs.
(C) Western blot detected the level of active β-catenin in HCT116 cells with the treatment of different concentration of recombinant human RSPO4 protein for 6 hrs.

Supplementary Figure 4. LGR4/5 and ZNRF3/RNF43 are required for RSPO4-induced suppression of Wnt/β-catenin signaling.
(A) Screening of *LGR4*, *LGR5*, *RNF43* and *ZNRF3* was performed in cancer cell lines by RT-PCR. After 48 hrs transcfection of empty vector and *RSPO4*, cells were harvested for RT-PCR analysis.
(B) Time course of changes in β-catenin following treatment of human recombinant RSPO4 protein in HCT116 and KYSE150 cells. The cells were stimulated with RSPO4 protein (100 ng/ml) for 0-6 hrs and collected for Western blot.

(C) Time course of changes in mRNA expression of *LGR4*, *LGR5*, *ZNRF3*, *RNF43 and c-MYC* detected by RT-PCR at different time points with treatment of human recombinant RSPO4 protein.

Supplementary Table 1. Primers used for the detection of *RSPO4* expression and promoter CpG methylation

Type	Primers	Sequence (5'-3')	Length (bp)
RT-PCR	RSPO4F	TGGACATGCTCGCCCTGAAC	255
	RSPO4R	TGAAGCAGCTCTCACAAGTG	
MSP	RSPO4-m4	GACGACACGACGAACGCG	164
	RSPO4-m7	GATTTTCGTTTTTTTTTCGCGC	
	RSPO4-u4	CAACAACAACACAACAAACACA	170
	RSPO4-u7	GTGATTTTTGTTTTTTTTTTGTGT	
Multiplex Genomic PCR	RSPO4F	TGGACATGCTCGCCCTGAAC	307
	RSPO4-Int1R	CTGCTGACCATGCGGCTAC	
	RSPO4-Int2F2	CAAGCAAGTCACTCGCTCTC	263
	RSPO4R	TGAAGCAGCTCTCACAAGTG	
	GAPDHInt7F	GCCTCACTCCTTTTGCAGAC	156
	GAPDH33	GATGACCTTGCCCACAGCCT	
BGS	RSPO4-BGS3	GGGGGAGGGAAAGAAAATTAT	565
	RSPO4-BGS4	CAACCACCCCTTATACCTTA	

Supplementary Table 2. Semi-quantitative RT-PCR primers for genes regulated by *RSPO4* expression

Gene	Primers	Sequence (5’-3’)	Length (bp)
c-MYC	MYCF	CTCTCCGTCCTCGGATTCTC	211
	MYCR	GCCTCCAGCAGAAGGTGATC	
LGR4	LGR4F	TAGGGCTGCTCTGCTTCCTC	306
	LGR4R	GTTCTTTCAACCCAGACAAG	
LGR5	LGR5F	AGGTCTGGTGTGTTGCTGAG	250
	LGR5R	CTCCCTTGGGAATGTATGTC	
RNF43	RNF43F	AGCGGTGGAGTCTGAAAGAT	207
	RNF43R	AATCCAGGCTCCAGATTGTC	
ZNRF3	ZNRF3FN	AGGAGTGGTGAAGCTGGAAC	329
	ZNRF3RN	AGGAGACCACGACGAAGAAAG	
GAPDH	GAPDH33	GATGACCTTGCCCACAGCCT	302
	GAPDH55	ATCTCTGCCCCCTCTGCTGA	

Supplementary Table 3. The primers used for plasmid construction of *RSPO4* and its mutants in this study

No.	Primers	Sequence (5'-3')
Wild-type	RSPO4-F-KpnI-FLAG	GGGGTACCGCCACCATGGATTACAAGGATGACGACGATAAGCGGGCGC CACTCTGCCTG
	RSPO4-R-BamHI-V5	CGGGATCCCTACGTAGAATCGAGACCGAGGAGAGGGTTAGGGATAGGCT TACCGGGCTGCAGGCCGGGCTG
R60A/Q65A	RSPO4-R60A/Q65A-F	CTTCCTGTTCATCCGCGCAGAAGGCATCCGCGCATACGGCAAGTGCCTG
	RSPO4-R60A/Q65A-R	CAGGCACTTGCCGTATGCGCGGATGCCTTCTGCGCGGATGAACAGGAAG
F99A/F103A	RSPO4-F99A/F103A-F	CACTTGTGAGAGCTGCGCAAGCCAGGACGCATGCATCCGGTGCAAGAG
	RSPO4-F99A/F103A-R	CTCTTGCACCGGATGCATGCGTCCTGGCTTGCGCAGCTCTCACAAGTG
ΔTSP	RSPO4-ΔTSP-F1	GAGTGTGAACTGGGTCCCACACACAATGGAAAGACCGAGGCTGGCCGGGCTG
	RSPO4-ΔTSP-R1	CAGCCCGGCCAGCCTCGGTCTTTCCATTGTGTGTGGGACCCAGTTCACACTC
	RSPO4-ΔTSP-F2n	ACCTGCCAGGTGCTTTCT CCCATCCAGAGGCCCTGC
	RSPO4-ΔTSP-R2n	GCAGGGCCTCTGGATGGG AGAAAGCACCTGGCAGGT
ΔTSP/BR	RSPO4-ΔBR-Rn-BamHI-V5	CGGGATCCCTACGTAGAATCGAGACCGAGGAGAGGGTTAGGGATAGGCTTAC CGCAGGGCCTCTGGATG

Supplementary Table 4. Relationship between *RSPO4* methylation and clinicopathologic features of patients with Brain LGG (TCGA, Firehose Legacy)

Clinical characteristic	*RSPO4* methylation		*P*-value
	No (n=435)	Yes (n=75)	
Gender			0.594
Female	191	36	
Male	244	39	
Diagnosis Age (years)			**0.000**
<=20	8	1	
>20 & < 60	383	49	
>= 60	44	25	
Histological Type			0.342
Anaplastic Astrocytoma	110	20	
Anaplastic Oligoastrocytoma	61	17	
Astrocytoma	57	6	
Diffuse Glioma	1	0	
Oligoastrocytoma	113	15	
Oligodendroglioma	94	17	
Neoplasm Histologic Grade			**0.034**
G2	217	27	
G3	217	48	
Karnofsky Performance Score			**0.000**
40 - 70	24	14	
80 - 100	223	32	
Ethnicity			**0.004**
American Indian or Alaska Native	0	1	
Asian	5	3	
Black or African American	15	6	
White	407	63	
Tumor size (cm)			0.632
<= 0.5	5	0	
>0.5 & <1.5	123	22	
>= 1.5	58	11	
Primary Tumor Laterality			0.563
Left	212	35	
Midline	5	2	

Right	213	38	
Asthma History			**<u>0.016</u>**
No	300	42	
Yes	14	7	
Headache History			0.146
No	243	49	
Yes	139	19	

Supplementary Table 5. Somatic mutations of *RSPO4* in multiple cancer types

Sample ID	Cancer Study	AA change	Type	Copy	COSMIC	PolyPhen-2	Ref.
C141	CRC	L7M	Missense	Heter.	1	Probably Damaging	[1]
HCC134	Liver	A14S	Missense	Heter.	1	Benign	COSMIC
HCC134T	Liver	A14S	Missense	Heter.	1	Benign	COSMIC
ESO-161	Esophagus	C35F	Missense	NA	1	Probably Damaging	TCGA
T3225	CRC	I39T	Missense	NA	1	Benign	[2]
MEL-Ma-Mel-102	Melanoma	G46D	Missense	NA	NA	Probably Damaging	[3]
9266982	nccRCC	T49AfsX176	Frameshift	NA	NA	Damaging	[4]
TCGA-CG-5721-01	Stomach	T49I	Missense	Diploid	1	Probably Damaging	[5]
2_PRE-TREATMENT	Melanoma	R59C	Missense	Homo.	NA	Benign	[6]
2_RESISTANT	Melanoma	R59C	Missense	Homo.	NA	Benign	[6]
TCGA-28-5209-01	GBM	R60Q	Missense	Diploid	1	Probably Damaging	TCGA
H081665	Liver	R60L	Missense	Diploid	1	Probably Damaging	[7]
CSCC-56-T	Skin	Y66X	Nonsense	Heter.	1	Damaging	[8]
LUAD-RT-S01711	LUAD	G67S	Missense	Diploid	1	Probably Damaging	[9]
S02378	LUSC	P75R	Missense	NA	NA	Benign	[10]
TCGA-17-Z043-01	LUAD	P75H	Missense	NA	1	Benign	COSMIC
TCGA-A3-3320-01	ccRCC	G76A	Missense	Diploid	1	Probably Damaging	[11]
MEL-Ma-Mel-53	Melanoma	R81S	Missense	NA	NA	Probably Damaging	[3]
TCGA-AX-A0J1-01	Uterine	R87M	Missense	Diploid	1	Possibly Damaging	[12]
TCGA-BR-8687-01	Stomach	K90Q	Missense	Gain	1	Probably Damaging	[5]
TCGA-FP-8211-01	Stomach	D102E	Missense	Diploid	2	Probably Damaging	[5]
TCGA-55-1596-01	LUAD	D102N	Missense	Gain	2	Probably Damaging	[13]
PACA-86-T	Pancreas	K117X	Nonsense	NA	2	Damaging	[14]
8044841	Pancreas	K117X	Nonsense	NA	NA	Damaging	COSMIC
TCGA-AA-A01D-01	CRC	T121I	Missense	Gain	NA	Probably Damaging	[15]
TCGA-06-0124-01	GBM	P123L	Missense	Diploid	4	Probably Damaging	TCGA
TCGA-EE-A3J7-06	Melanoma	P123L	Missense	Gain	4	Probably Damaging	TCGA
TCGA-CG-4306-01	Stomach	P123L	Missense	Diploid	4	Probably Damaging	[5]
TCGA-97-7938-01	LUAD	L127F	Missense	Diploid	1	Possibly Damaging	[13]
TCGA-OR-A5KP-01	ACC	P143T	Missense	ShallowDel	NA	Possibly Damaging	TCGA

OSCC-GB_00700111	Upper AT	W144X	Nonsense	NA	NA	Damaging	COSMIC
S01023	LUSC	W147C	Missense	NA	NA	Probably Damaging	[10]
TCGA-IB-7651-01	Pancreas	S159L	Missense	Diploid	1	Benign	TCGA
MEL-Ma-Mel-63	Melanoma	S159L	Missense	NA	1	Benign	[3]
HDC101	CRC	R166Q	Missense	Heter.	1	Probably Damaging	[16]
TCGA-A6-2675-01	CRC	R166Q	Missense	NA	1	Probably Damaging	COSMIC
3885	Liver	R166GfsX60	Frameshift	NA	1	Damaging	[17]
MEL-Ma-Mel-35	Melanoma	R172W	Missense	NA	NA	Probably Damaging	[3]
TCGA-HT-7616-01	Glioma	E177G	Missense	Diploid	1	Probably Damaging	TCGA
TCGA-CK-6746-01	CRC	Q182H	Missense	NA	1	Possibly Damaging	COSMIC
HCA46	CRC	V183L	Missense	Heter.	1	Benign	[16]
SC_9023	Prostate	R211Q	Missense	Gain	NA	Benign	[18]
TCGA-CA-5254-01	CRC	R214C	Missense	NA	1	Probably Damaging	COSMIC
TCGA-D5-6540-01	CRC	R214C	Missense	NA	1	Probably Damaging	COSMIC
T3266	CRC	R214QfsX>21	Frameshift	NA	1	Damaging	[2]
S01297	LUSC	R222S	Missense	NA	NA	Benign	[10]
OSCC-GB_00960111	Upper AT	R228H	Missense	NA	1	Benign	COSMIC
AOCS-090-1-0	Ovary	Q233K	Missense	NA	1	Benign	COSMIC

AA, amino acid; ACC, Adrenocortical Carcinoma; GBM, Glioblastoma Multiforme; CRC, Colorectal Cancer; ccRCC, Clear Cell Renal Cell Carcinoma; LUAD, Lung Adenocarcinoma; nccRCC, non-clear Cell Renal Cell Carcinoma; LUSC, Lung Squamous Cell Carcinoma; Upper AT, upper aero-digestive tract. Data extracted from cBioPortal (http://www.cbioportal.org/) and COSMIC (http://cancer.sanger.ac.uk/cosmic).

Supplementary Table 6. Germline mutations of *RSPO4* in human genetic diseases

No.	Mutation	Domain	Genetic disease	Mutation pattern	Origin	Ref.
1	c.C178T (p.R60W)	FU1	Anonychia	Homo.	Pakistani	[19]
2	c.G353A (p.C118Y)	FU2	hyponychia	Homo.	Pakistani	
3	c.C18A (p.C6X)	SP	Anonychia	Homo.	Pakistani	[20]
4	c.G199C (p.G67R)	FU1	Anonychia	Homo.	Pakistani	[21]
5	c.C190T (p.R64C)	FU1	Anonychia	Homo.	Turkish	[22]
6	c.C301T (p.E101X)	Truncating	Anonychia	Homo.	Kazakh	
7	IVS2-1G>A	Truncating	Anonychia	Homo.	Pakistani	[23]
8	c.-9-p17del26	Truncating	Anonychia	Homo.	Pakistani	
9	c.G3A (p.M1I)	SP	Anonychia	Homo.	Pakistani	
10	c.92_93insG (p.L31fs)+ c.G218A (p.C73Y)	Truncating	Anonychia	Homo.	German	[24]
11	c.95_110del16 (p.G32AfsX189)	Truncating	Anonychia	Homo.	Indian	[25]
12	p.C118Y	FU2	hyponychia	Homo.	Pakistani	
13	c.-9-+17del26	Truncating	Hyponychia	Homo.	Pakistani	
14	IVS1+1G>A	Truncating	Hyponychia	Homo.	Pakistani	
15	c.A194G (p.Q65R)	FU1	Anonychia	Homo.	Finnish	
16	p.C107R	FU2	Anonychia	Homo.	Irish	
17	IVS1-1G>A+p.C95F	Truncating	Anonychia	Compound heter.	UK	
18	p.C95F+p.C107R	FU2	Anonychia	Compound heter.	UK	

SP, signal peptide

References:

1. Tahara T, Yamamoto E, Madireddi P, Suzuki H, Maruyama R, Chung W, et al. Colorectal carcinomas with CpG island methylator phenotype 1 frequently contain mutations in chromatin regulators. Gastroenterology. 2014; 146: 530-38 e5.
2. Giannakis M, Hodis E, Jasmine Mu X, Yamauchi M, Rosenbluh J, Cibulskis K, et al. RNF43 is frequently mutated in colorectal and endometrial cancers. Nature genetics. 2014; 46: 1264-6.
3. Hodis E, Watson IR, Kryukov GV, Arold ST, Imielinski M, Theurillat JP, et al. A landscape of driver mutations in melanoma. Cell. 2012; 150: 251-63.
4. Durinck S, Stawiski EW, Pavia-Jimenez A, Modrusan Z, Kapur P, Jaiswal BS, et al. Spectrum of diverse genomic alterations define non-clear cell renal carcinoma subtypes. Nature genetics. 2015; 47: 13-21.
5. Cancer Genome Atlas Research N. Comprehensive molecular characterization of gastric adenocarcinoma. Nature. 2014; 513: 202-9.
6. Wagle N, Van Allen EM, Treacy DJ, Frederick DT, Cooper ZA, Taylor-Weiner A, et al. MAP kinase pathway alterations in BRAF-mutant melanoma patients with acquired resistance to combined RAF/MEK inhibition. Cancer discovery. 2014; 4: 61-8.
7. Ahn SM, Jang SJ, Shim JH, Kim D, Hong SM, Sung CO, et al. Genomic portrait of resectable hepatocellular carcinomas: implications of RB1 and FGF19 aberrations for patient stratification. Hepatology. 2014; 60: 1972-82.
8. Pickering CR, Zhou JH, Lee JJ, Drummond JA, Peng SA, Saade RE, et al. Mutational landscape of aggressive cutaneous squamous cell carcinoma. Clinical cancer research : an official journal of the American Association for Cancer Research. 2014; 20: 6582-92.
9. Imielinski M, Berger AH, Hammerman PS, Hernandez B, Pugh TJ, Hodis E, et al. Mapping the hallmarks of lung adenocarcinoma with massively parallel sequencing. Cell. 2012; 150: 1107-20.
10. George J, Lim JS, Jang SJ, Cun Y, Ozretic L, Kong G, et al. Comprehensive genomic profiles of small cell lung cancer. Nature. 2015; 524: 47-53.
11. Cancer Genome Atlas Research N. Comprehensive molecular characterization of clear cell renal cell carcinoma. Nature. 2013; 499: 43-9.
12. Cancer Genome Atlas Research N, Kandoth C, Schultz N, Cherniack AD, Akbani R, Liu Y, et al. Integrated genomic characterization of endometrial carcinoma. Nature. 2013; 497: 67-73.
13. Cancer Genome Atlas Research N. Comprehensive molecular profiling of lung adenocarcinoma. Nature. 2014; 511: 543-50.
14. Biankin AV, Waddell N, Kassahn KS, Gingras MC, Muthuswamy LB, Johns AL, et al. Pancreatic cancer genomes reveal aberrations in axon guidance pathway genes. Nature. 2012; 491: 399-405.
15. Cancer Genome Atlas N. Comprehensive molecular characterization of human colon and rectal cancer. Nature. 2012; 487: 330-7.
16. Mouradov D, Sloggett C, Jorissen RN, Love CG, Li S, Burgess AW, et al. Colorectal cancer cell lines are representative models of the main molecular subtypes of primary cancer. Cancer research. 2014; 74: 3238-47.
17. Jhunjhunwala S, Jiang Z, Stawiski EW, Gnad F, Liu J, Mayba O, et al. Diverse modes of genomic alteration in hepatocellular carcinoma. Genome biology. 2014; 15: 436.
18. Robinson D, Van Allen EM, Wu YM, Schultz N, Lonigro RJ, Mosquera JM, et al. Integrative clinical genomics of advanced prostate cancer. Cell. 2015; 161: 1215-28.
19. Khan TN, Klar J, Nawaz S, Jameel M, Tariq M, Malik NA, et al. Novel missense mutation in the RSPO4 gene in congenital hyponychia and evidence for a polymorphic initiation codon (p.M1I). BMC medical genetics. 2012; 13: 120.
20. Wasif N, Ahmad W. A novel nonsense mutation in RSPO4 gene underlies autosomal recessive congenital anonychia in a Pakistani family. Pediatric dermatology. 2013; 30: 139-41.
21. Chishti MS, Kausar N, Rafiq MA, Amin M, Ahmad W. A novel missense mutation in RSPO4 gene underlies autosomal recessive congenital anonychia in a consanguineous Pakistani family. The British journal of dermatology. 2008; 158: 621-3.

22. Bruchle NO, Frank J, Frank V, Senderek J, Akar A, Koc E, et al. RSPO4 is the major gene in autosomal-recessive anonychia and mutations cluster in the furin-like cysteine-rich domains of the Wnt signaling ligand R-spondin 4. The Journal of investigative dermatology. 2008; 128: 791-6.
23. Ishii Y, Wajid M, Bazzi H, Fantauzzo KA, Barber AG, Blaydon DC, et al. Mutations in R-spondin 4 (RSPO4) underlie inherited anonychia. The Journal of investigative dermatology. 2008; 128: 867-70.
24. Bergmann C, Senderek J, Anhuf D, Thiel CT, Ekici AB, Poblete-Gutierrez P, et al. Mutations in the gene encoding the Wnt-signaling component R-spondin 4 (RSPO4) cause autosomal recessive anonychia. American journal of human genetics. 2006; 79: 1105-9.
25. Blaydon DC, Ishii Y, O'Toole EA, Unsworth HC, Teh MT, Ruschendorf F, et al. The gene encoding R-spondin 4 (RSPO4), a secreted protein implicated in Wnt signaling, is mutated in inherited anonychia. Nature genetics. 2006; 38: 1245-7.
